# Acceleration of Protein Degradation by 20S Proteasome-Binding Peptides Generated by In Vitro Artificial Evolution

**DOI:** 10.3390/ijms242417486

**Published:** 2023-12-14

**Authors:** Yunhao Zhu, Kaishin Shigeyoshi, Yumiko Hayakawa, Sae Fujiwara, Masamichi Kishida, Hitoshi Ohki, Tomohisa Horibe, Masafumi Shionyu, Tamio Mizukami, Makoto Hasegawa

**Affiliations:** 1 Graduate School of Bioscience, Nagahama Institute of Bio-Science and Technology, 1266 Tamura-cho, Nagahama 526-0829, Japan; 2 Modality Research Laboratories, Biologics Division, Daiichi Sankyo Co., Ltd., 1-2-58, Hiromachi, Shinagawa-ku, Tokyo 140-8710, Japan; 3 Frontier Pharma Inc., 1281-8 Tamura, Nagahama 526-0829, Japan

**Keywords:** directed evolution, peptides, photoaffinity labeling, proteasome, protein degradation

## Abstract

Although the 20S core particle (CP) of the proteasome is an important component of the 26S holoenzyme, the stand-alone 20S CP acts directly on intrinsically disordered and oxidized/damaged proteins to degrade them in a ubiquitin-independent manner. It has been postulated that some structural features of substrate proteins are recognized by the 20S CP to promote substrate uptake, but the mechanism of substrate recognition has not been fully elucidated. In this study, we screened peptides that bind to the 20S CP from a random eight-residue pool of amino acid sequences using complementary DNA display an in vitro molecular evolution technique. The identified 20S CP-binding amino acid sequence was chemically synthesized and its effects on the 20S CP were investigated. The 20S CP-binding peptide stimulated the proteolytic activity of the inactive form of 20S CP. The peptide bound directly to one of the α-subunits, opening a gate for substrate entry on the α-ring. Furthermore, the attachment of this peptide sequence to α-synuclein enhanced its degradation by the 20S CP in vitro. In addition to these results, docking simulations indicated that this peptide binds to the top surface of the α-ring. These peptides could function as a key to control the opening of the α-ring gate.

## 1. Introduction

The ubiquitin proteasome system is the major proteolytic pathway that maintains intracellular protein homeostasis. It plays a central role in a wide range of physiological processes, including cell cycling, gene expression, signal transduction, immune responses, stress responses, protein quality control, and others [1,2]. In eukaryotic cells, the 26S proteasome holoenzyme degrades ubiquitin-modified proteins, and the 20S core particle (CP) degrades unfolded proteins into short peptides of varying length [3,4].

In the 26S holoenzyme, the 19S regulatory particle (19S RP) is responsible for the recognition, unfolding, and sorting of ubiquitinated proteins into the 20S CP [5]. The 19S RP binds to one or both sides of the 20S CP to form the 26S holoenzyme. The 20S CP contains threonine protease subunits within four stacked rings, with three catalytic subunits (β5, β2, β1) in the inner two β-rings, each with chymotrypsin-like (CT-L), trypsin-like (T-L), and polyglutamate hydrolysis (PGPH) protease activities [6,7]. The outer α-ring does not possess protease activity, but controls access to the proteolytic sites in the inner chamber of the 20S CP via an allosterically regulated gate opening/closing mechanisms [8]. Cryogenic electron microscopy analyses have revealed several distinct conformational forms of the 26S holoenzyme, as well as details of the mechanisms of gate regulation; the six ATPases (Rpt-1-6) at the base of the 19S RP lid are composed of six ATPases with C-terminal HbYX motifs (a hydrophobic amino acid, a tyrosine, and any other amino acid) [9,10]. After traversing the channel of the 19S RP lid, unfolded substrates pass through the gate on the α-ring and cross the β-annulus to access the proteolytic chamber of the 20S CP.

Although the 20S CP is an integral part of the 26S holoenzyme, the stand-alone 20S CP is believed to function independently by acting directly on disordered or oxidized/damaged proteins [11]. However, our understanding of the proteolytic mechanism of the stand-alone 20S CP lags behind that of the 26S holoenzyme. Since the free form of the 20S CP is in a dynamic equilibrium state with the 26S holoenzyme, in which approximately half of proteasomes in most eukaryotic cells are in the free 20S CP state [12,13], ubiquitin-independent proteolysis by the 20S CP may occur alongside degradation by the 26S holoenzyme. Furthermore, since the ratio of the 20S CP to the 26S holoenzyme varies with cell type and conditions, the equilibrium between the two forms may be part of an adaptive response to cellular demand [2].

Interestingly, the 20S CP is more resistant to oxidative damage than the 26S holoenzyme [14,15]. Various reports suggest that under cellular stresses such as oxidative stress and hypoxic stress, the 20S CP may serve as an emergency proteasome and contribute to survival under proteotoxic conditions [11]. Under hypoxia, the intracellular ratio of the 20S CP to the 26S holoenzyme changes significantly, with free 20S CP becoming the predominant proteasome form. This form has been shown to enhance cellular tolerance to hypoxia by efficiently degrading damaged proteins [16]. Substrate proteins for the stand-alone 20S CP have intrinsically disordered regions (IDRs) longer than 30 amino acids that lack an ordered three-dimensional structure, which makes them intrinsically sensitive to proteolysis [17]. Although >40% of human protein-coding genes contain IDRs, not all IDR-containing proteins are degraded by the 20S CP; hence, stand-alone 20S CP should be able to distinguish specific substrate proteins. Certain interactions between the free 20S CP and IDR structures appear to open the gates in the α-rings, facilitating the degradation of proteins with IDR structures [4]. However, which segmental structures of substrates interact with the α-rings of the free 20S CP form remains unknown.

To date, artificial peptides derived from the HIV-1 trans-activator of transcription protein (Tat) [18], a cathelin-like proline-arginine-rich peptide (PR-peptide) [19], proteasome-activating peptide 1 (PAP1) [20], the C-terminal region of Rpt2/3/5 [21], and cell-penetrating peptide octa-arginine have been reported to interact with the 20S CP [22]. The surfactant sodium dodecyl sulfate (SDS) is known to activate substrate degradation by loosening the gate structure of the latent 20S CP [23]. In addition, recognition chemical moieties linked to (Boc_3_)-protected arginine bind to and activate the 20S CP, inducing the degradation of target proteins [24]. Since these peptides and compounds regulate the 20S CP in an allosteric manner, they may share a common mechanism with the gate-control mechanism of free 20S CP when it recognizes specific substrate proteins containing IDRs.

**Figure 1:**
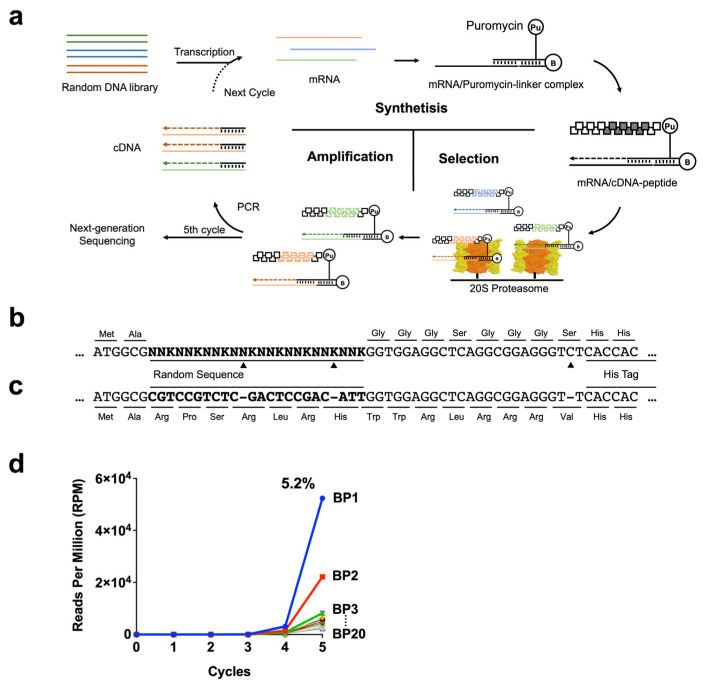
Generation of 20S proteasome-binding peptides using the cDNA display method. (**a**) Overview of the cDNA display method. (**b**) Designed DNA sequence in the cDNA display method. N = A, C, G, and T; K = G and T. The black triangles indicate bases that were deleted during the convergence process and caused the frameshift. (**c**) Convergent DNA sequence and amino acid sequence of 20S-BP1. (**d**) Read numbers of identical sequences per 10^6^ reads (RPM) in each cycle analyzed by next-generation sequencing (NGS).

In the present study, the random screening of amino acid sequences interacting with the outer surface of the 20S CP was used to identify 20S-interacting peptides, providing tools to elucidate the interactions of IDR-containing proteins with the 20S CP involved in proteasome control. The complementary DNA (cDNA) display method was used to comprehensively explore peptides binding to the 20S CP (Figure 1a) [25,26]. The cDNA display approach is an in vitro molecular evolution technique that selects from random sequence library peptides that bind with high affinity and specificity to a given target. Using puromycin linker DNA, mRNA/cDNA peptide complexes can be generated. From random peptide libraries linked with corresponding nucleic acid sequences, amino acid sequences with affinity for the 20S CP are selected and amplified via DNA. The cycle is repeated several times to obtain peptides with high affinity for the purified 20S CP. Protease activity assays showed that the selected peptide inhibited 20S CT-L activity activated by SDS. Furthermore, the peptide promoted the proteolytic activities of latent 20S CP without SDS treatment. The 20S CP-binding peptide with an additional cell-penetrating sequence, the antennapedia peptide [27], increased intracellular proteasome activity, eventually resulting in cell death. In addition, the effects of these peptides attached to a substrate protein α-synuclein (α-Syn) on 20S CP proteolysis were examined, and the peptide promoted degradation when added to the C-terminus. Some proteins known to be native substrates of free 20S CP have interaction sites within their own amino acid sequences that open the α-gate and guide them to the 20S chamber [28]. The interacting peptides obtained from the random sequence pool in this study may have properties similar to the interacting amino acid sequences of such native substrates of the 20S CP.

## 2. Results and Discussion

### 2.1. Generation of 20S-Interacting Peptides Using the cDNA Display Method

In the first cycle of library screening using the cDNA display method, a complex peptide–mRNA/cDNA library was generated from a DNA pool containing random codons equivalent to eight amino acid residues (Figure 1b). The fraction bound to nanomagnetic beads carrying human 20S CP was isolated from the library, and DNA bound to peptides was amplified by PCR. This cycle was repeated five times. Next, DNA libraries from each cycle were analyzed by next-generation sequencing (NGS). In DNA pools from advanced cycling, the original sequence in each DNA pool was replaced by a frameshift sequence during each cycle, decreasing to 40% by the fifth cycle. In the most enriched DNA sequence, two bases inside the random segment (NNK repeats) were deleted, as was one base inside the linker-coding segment (Figure 1c). The amino acid sequence encoded by the most enriched random segment was that with seven residues: Arg-Pro-Ser-Arg-Leu-Arg-His. The amino acid sequence of the linker-coding segment was altered by frameshift from the original Gly-Gly-Gly-Ser-Gly-Gly-Gly-Ser to Trp-Trp-Arg-Leu-Arg-Arg-Arg-Val. The enrichment of convergent sequences began from the fourth round, with the most enriched sequence accounting for 5.2% of the total (Figure 1d).

### 2.2. Proteasome Inhibition by 20S-BP1 and Its Analogs

**Figure 2:**
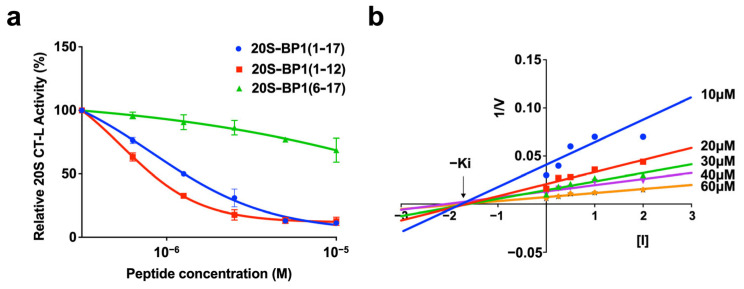
Profiles of 20S CT-L activity inhibition by 20S-BP1 peptides. (**a**) Inhibition curves for synthetic peptides 20S-BP1(1-17) (blue closed triangles), 20S-BP1(1-12) (red closed rectangles), and 20S-BP1(6-17) (green closed circles) against chymotrypsin-like activity (CT-L) of the 20S CP. Means ± SD, *n* = 3. (**b**) Dixon plot analysis of inhibition of CT-L activity by 20S-BP1(1-12). The substrate concentrations are as follows: 10 μM (blue closed circles), 20 μM (red closed rectangles), 30 μM (green closed rectangles), 40 μM (violet closed invert triangles), and 60 μM (orange closed diamonds).

The most enriched sequence was chemically synthesized as a peptide consisting of 17 amino acid residues, Met-Ala-Arg-Pro-Ser-Arg-Leu-Arg-His-Trp-Trp-Arg-Leu-Arg-Arg-Arg-Val, named 20S-BP1(1-17). The peptide had an IC_50_ value of 1.35 ± 0.21 μM against the CT-L activity of purified human 20S CP treated with 0.03% SDS (Figure 2a and Table 1). The N-terminal region peptide, 20S-BP1(1-12), inhibited CT-L activity with an IC_50_ of 0.93 ± 0.30 μM, while the C-terminal region peptide, 20S-BP1(6-17), showed no inhibition at a concentration of 10 μM (Figure 2a). The mode of inhibition of CT-L activity by 20S-BP1(1-12) was evaluated with a Dixon plot analysis (Figure 2b). The regression lines for the plots at each peptide concentration intersected with the x-axis, indicating a noncompetitive mode of inhibition for 20S-BP1(1-12). The inhibition constant (K_i_) value was 1.7 ± 0.1 μM, which was almost equal to the IC_50_ value. These results indicate that the interaction site is contained in a twelve-residue region that includes the seven-amino acid sequence Arg-Pro-Ser-Arg-Leu-Arg-His enriched from the random sequence pool and the three-amino acid sequence Trp-Trp-Arg generated by frameshift of the linker-coding segment (Figure 1c).

**Figure 3:**
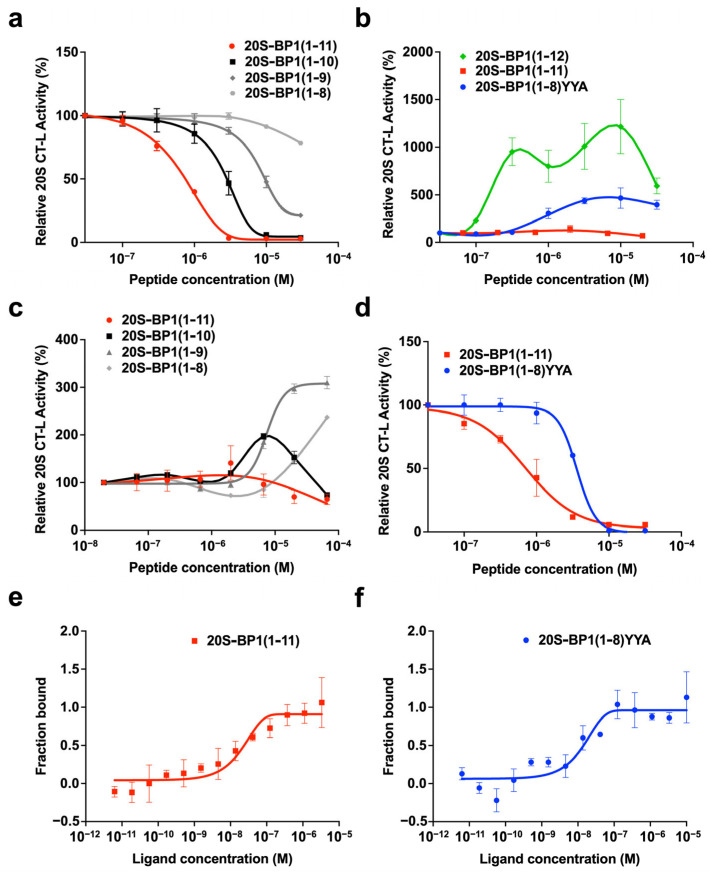
Effects of 20S-BP1 peptides on the 20S CP. Inhibition (with 0.03% SDS) (**a**) and promotion (without SDS) (**c**) of the CT-L activity of the 20S CP by 20S-BP1(1-11) and short analogs with C-terminal deletions. Promotion (without SDS) (**b**) and inhibition (with 0.03% SDS) (**d**) of CT-L activity of the 20S CP by 20S-BP1(1-12), 20S-BP1(1-11), and 20SP-BP1(1-8)YYA. Results are presented as relative activities based on the amount of fluorescent substrate (Suc-LLVY-AMC) hydrolyzed by 20S CP (100 μg) in 60 min. Means ± SD, *n* = 3. (**e**,**f**) MST binding assays. (**e**) Affinity of 20S-BP1(1-11) and (**f**) 20S-BP1(1-8)YYA peptides for the 20S CP. Binding affinity is plotted as fraction bound with values from 0 to 1 (0 = unbound, 1 = bound) against a range of peptide concentrations from 10^−11^ to 10^−5^ M. The K_D_ of the interaction was determined by fitting the data. Means ± SD, *n* = 3.

We focused on 20S-BP1(1-12) and examined the relationship between the structure and inhibition 20S CP activity (Figure 3, Table 1, and Supplementary Information Figure S1). The deletion of the C-terminal Arg^12^ residue had no effect, but the deletion of the Trp^10^-Trp^11^ sequence increased the IC_50_ value, and further loss of Arg^8^-His^9^ resulted in no inhibitory activity (Figure 3a). Furthermore, when the chain length was shortened from the N-terminus, 20S-BP1(3-12) showed no inhibitory activity. However, 20S-BP1(4-12) and 20S-BP1(5-12), in which the N-terminal region was further shortened, recovered the inhibitory activity, and had the lowest IC_50_ value (0.82 μM). The significant loss of inhibitory activity due to the presence of Arg^3^ exposed at the N-terminus (20S-BP1(3-12)) could indicate that the side chain of this basic amino acid residue potentially adopts a conformation that interferes with the binding of the peptide to the 20S CP. Furthermore, 20S-BP1(6-12) and 20S-BP1(7-12) had IC_50_ values approximately two-fold higher than that of 20S-BP1(5-12), suggesting that Ser^5^ of 20S-BP1(5-12) increases its affinity for the 20S CP. Throughout, 20S-BP1(5-12) showed the most potent inhibitory activity, suggesting that this eight-residue amino acid sequence possesses the minimum structure required for binding to the 20S CP. On the other hand, His^9^-Trp^10^-Trp^11^ in the C-terminal aromatic amino acid region may interact directly, since its deletion greatly impaired the inhibitory effect. The substitution of this region with Ala one residue at a time did not result in a large decrease in inhibitory activity, but a single substitution of Met^1^, Pro^4^, Ser^5^, Leu^7^, His^9^, Trp^10^, or Trp^11^ increased the IC_50_ value 2–5-fold (Table 1). The results suggest that interactions between hydrophobic amino acid side chains provide the basis for the affinity between the peptide and the 20S CP.

### 2.3. Proteasome Stimulation by 20S-BP1(1-12) and Its Analogs

Some peptides are known to inhibit 20S CP activity in vitro in the presence of 0.03% SDS but activate it in the absence of SDS [18,19,20,21,22]. Herein, we examined the activating properties of 20S-BP1 for the CT-L activity of latent 20S CP without SDS treatment. The 20S-BP1(1-12) profile revealed activation (Figure 3b and Table 1), with an AC_max_ achieving 1215% activation at 10 μM (Table 1). In contrast, the profile of 20S-BP1(1-11), which lacked the C-terminal Arg, showed weak activation, with an AC_max_ of 149% activation at 100 μM. The activation profile of the short peptide showed that proteasome activity remained in the range of 107–310% (Table 1). The deletion of the C-terminal Trp^10^-Trp^11^ sequence restored the proteasome activity by up to 310%, in contrast to the inhibitory effect. Thus, the C-terminal location of these two Trp residues in the peptide may inhibit its ability to activate the proteosome (Figure 3c). Therefore, we attempted to replace the C-terminal three residues with the HbYX motif, a consensus sequence that opens the α-gate. The HbYX motif is located at the C-terminus of the six Rpt subunits, and Tyr-Tyr-Ala is the motif sequence of Rpt5.

Without SDS treatment, the 20S CP was activated with up to 467% activity by 10 μM 20S-BP1(1-8)YYA, followed by a decrease in effect, resulting in a bell-shaped curve (Figure 3b). The substituted peptide, 20S-BP1(1-8)YYA, inhibited the CT-L activity of 20S CP under 0.03% SDS treatment, with an IC_50_ value of 3.80 μM (Figure 3d). In the presence of SDS, the peptides exhibited the opposite effect of gate opening (i.e., inhibition of substrate degradation). This may be due to the binding of the peptide to the lysine pocket (K-pocket) in the vicinity of the gate, which prevents the SDS-induced loosening of the gate structure and restores it to its normal gate structure.

The replacement of the C-terminus of 20S-BP1 with the HbYX motif of other Rpt subunits, TYN (Rpt1), LYL (Rpt2), FYK (Rpt3), KPV (Rpt4), and LWK (Rpt6), also promoted 20S CP activation in the absence of SDS. However, the proteasome activity remained in the range of 183–313% (Table 1). Although the C-terminal HbYX motifs of Rpt2, Rpt3, and Rpt5 have been reported to open the gate by docking into a hydrophobic pocket on the axial face of the core particle [1], only the synthetic peptides in the C-terminal region of Rpt 5 (Rpt5-C) activated the 20S CP among Rpt2-C, Rpt3-C, and Rpt5-C (Table 1).

The titration profiles of 20S-BP1(1-12) and its analogs during activation showed a systematic decrease in efficacy beyond the concentration that achieved a significant concentration-dependent AC_max_, resulting in a bell-shaped curve. A similar phenomenon was reported when the HbYX motif was added to the proline- and arginine-rich peptide PR11, which interacts with the 20S CP [19]. Short PR11 peptide fragments also inhibited SDS-activated 20S CP, as did the 20S-BP1 peptide, but they mildly activated inactive 20S CP when they contained HbYX or other similar hydrophobic C-terminal extensions. These results suggest that 20S-BP1 and RP peptides may act on the proteasome through a common mechanism.

Interestingly, the sequence Met-Ala-Arg-Pro-Ser-Arg-Leu-Arg-His-Trp-Trp-Arg showed stronger activation of the 20S CP at lower concentrations than any 20S-BP1 analog or Rpt-C peptide (Table 1). However, those with the C-terminal Arg removed failed to activate and showed only the inhibition of the 20S CP. This suggests that the positive charge at the C-terminus may play an important role in promoting gate opening of the 20S CP. The Ser-Arg-Leu-Arg-His-Trp-Trp region was sufficient to inhibit CT-L activity of the 20S CP, but the extension of the N-terminus by four amino acid residues (Met-Ala-Arg-Pro) greatly increased CT-L activity. The N-terminal exposure of Arg^3^ (20S-BP1(3-12)) resulted in a loss of inhibitory activity (Table 1). On the other hand, the fact that this peptide retained 154% of its enhancing activity indicates that activation and inhibition may not operate via the same interaction mode. In previous reports, peptides rich in basic amino acids such as polyarginine, Tat and PR peptides have been reported to promote 20S CP activation [18,19,20]. The simultaneous introduction of hydrophobic (Met^1^) and basic amino acids (Arg^3^ and Arg^12^) into 20S-BP1 peptides may result in increased 20S CP activity for reasons other than their effects on their binding affinity for the 20S CP.

The binding of the substitution peptide 20S-BP1(1-11) to purified 20S CP was experimentally confirmed by microscale thermophoresis assays, with a dissociation constant (K_D_) of 20.1 ± 5.8 nM (Figure 3e). The K_D_ of 20S-BP1(1-8)YYA measured under the same conditions was 9.9 ± 4.8 nM (Figure 3f). These results suggest that the C-terminal Tyr-Tyr-Ala substitution of 20S-BP1(1-11) did not impair its affinity for the 20S CP.

### 2.4. Effect on the 26S Holoenzyme

**Figure 4:**
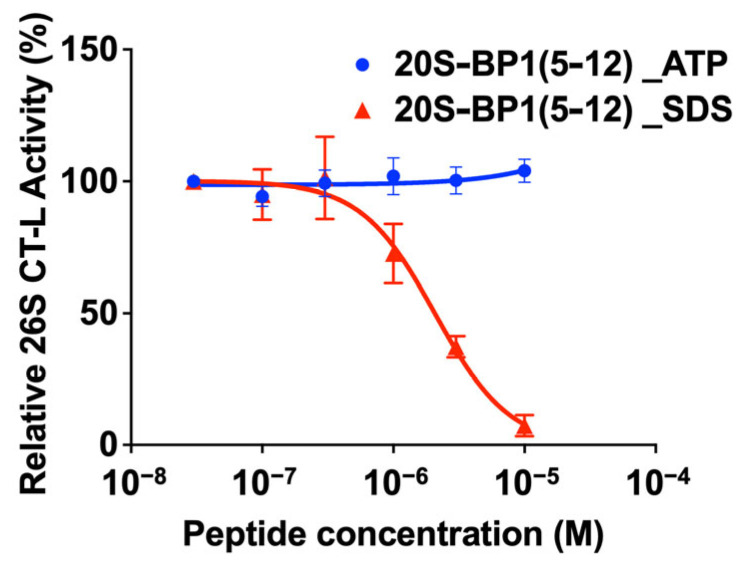
The 26S proteasome fluorometric substrate assay. Inhibition curve of 20S-BP1(5-12) against the 26S proteasome holoenzyme in the presence of ATP (blue closed circles) or 0.03% SDS (red closed triangles).

In the 26S holoenzyme, the binding of 19S RP to 20S CP is stabilized in the presence of ATP. Here, we used 20S-BP1(5-12), which showed the strongest inhibition of the 20S CP in the presence of SDS but two-fold activation in the absence of SDS (Table 1), to test its effect on the human 26S proteasome. The 20S-BP1(5-12) did not inhibit the CT-L activity of the 26S holoenzyme with 19S RP in the associated state in the presence of ATP, suggesting that it acts only on the 20S CP form (Figure 4). The 26S holoenzyme treated with 0.03% SDS had an inhibition profile of 20S-BP1(5-12), similar to that of the 20S CP. This is possibly because the 26S holoenzyme was dissociated by SDS to release the 20S CP. Similarly, no activating effect on the 26S proteasome was observed for 20S-BP1(1-12) (Figure S2).

### 2.5. Photoaffinity Labeling of 20S-BP1-Binding Subunits in the 20S CP

**Figure 5:**
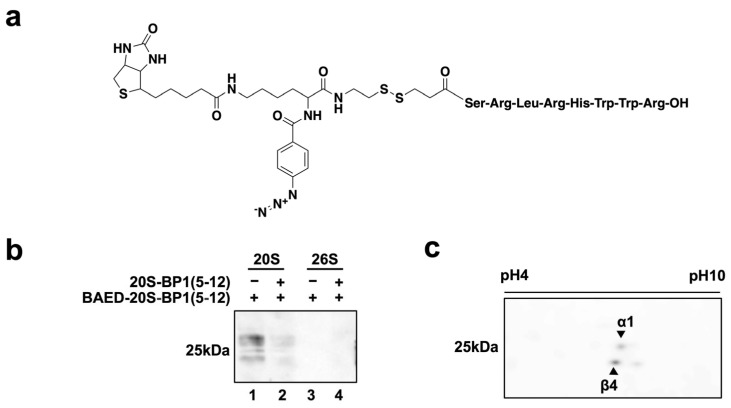
Photoaffinity labeling of the peptide binding site of proteasomes. (**a**) The biotinylated photoreactive peptide BAED-20S-BP1(5-12). (**b**) Labeling of purified proteasomes with BAED-20S-BP1(5-12). (**c**) 2D-PAGE electrophoresis images with photoaffinity labeling detection. Protein spots were identified by mass spectrometry after in-gel trypsin digestion as described in Section 3.

To clarify which subunit(s) of the 20S CP bind to 20S-BP1 peptides, we synthesized a peptide with a biotinylated photoreactive group, BAED. The presence of Arg^3^ weakened the inhibition of CT-L activity in the 20S CP (20S-BP1(3-12), Table 1). Furthermore, in photoaffinity labeling methods, it is generally important that the position of the photoreactive group is in close proximity to the direct receptor interaction site in the ligand. For these reasons, we selected 20S-BP1(5-12), a short peptide that showed sufficient inhibitory activity at low concentrations, as the peptide for attachment to the N-terminal BAED group (Figure 5a). BAED-20S-BP1(5-12) was crosslinked with the 20S CP in vitro by UV irradiation, and affinity-labeled 20S CP was separated and detected by one-dimensional (normal) SDS-polyacrylamide gel electrophoresis (PAGE) (Figure 5b, Lane 1) or two-dimensional SDS-PAGE (Figure 5c). The labeling of the 20S CP with the photoreactive peptide was reduced by competitive binding of the unmodified peptide 20S-BP1(5-12), and no chemiluminescence of the protein bands was detected in the absence of UV light (Figure 5b, Lane 2). On the other hand, the 26S holoenzyme showed no detectable labeling by the photoreactive peptide after UV irradiation (Figure 5b, Lanes 3 and 4), consistent with the effect of 20S-BP1(5-12) on enzyme activity (Figure 4). Two prominent labeled subunits were observed in the 2D electrophoresis images (Figure 5c). These Coomassie Brilliant Blue (CBB)-stained protein spots at the same positions were excised, subjected to in-gel enzyme digestion, and analyzed by liquid chromatography–mass spectrometry (LC-MS), which revealed that they corresponded to the α1 and β4 subunits (Table S1). Similar results were obtained in the affinity-labeled subunits of the 20S CP by BAED-20S-BP1(5-8)YYA (Figure S3). These results strongly suggest that the binding site of these 20S CP-activating peptides is around the K-pocket, the HbYX motif action site, of the α1 subunit. The β4 subunit is adjacent to the β5 subunit responsible for CT-L activity, and since the 20S-BP1 peptide can be a substrate for the 20S CP, it may have been detected because of affinity-labeling near the substrate binding site during the hydrolysis inside the 20S CP chamber.

Access to the internal cavity of the 20S CP is via a channel in the center of the α-ring [10,29]. In the quiescent ‘latent’ state, the channel is sealed by the entanglement of the extended N-termini of all seven α-subunits. When regulatory factors (19S/PA200/PA28) bind, these N-termini move and open or close depending on the functional state of the proteasome, and hence this region is called the ‘gate’ of the 20S CP. The lysine pocket (K-pocket) on the outer α surface binds to the HbYX motif at the C-terminus of most activators of the 20S CP, triggering gate opening. The Tat peptide with the HbYX motif in its C-terminus has been reported to induce gate opening of the 20S CP and to bind between α1 and α2 [18]. The peptides identified in this study may behave like the Tat peptide, in which the N-terminal region has affinity for the binding site near the gate, and the hydrophobic amino acid side chain in the C-terminal region inserts into the K-pocket on the 20S CP surface, triggering gate opening and facilitating substrate degradation.

### 2.6. Effect of 20S-BP1 Analogs with a Cell-Penetrating Peptide on Intracellular Proteasomes

**Figure 6:**
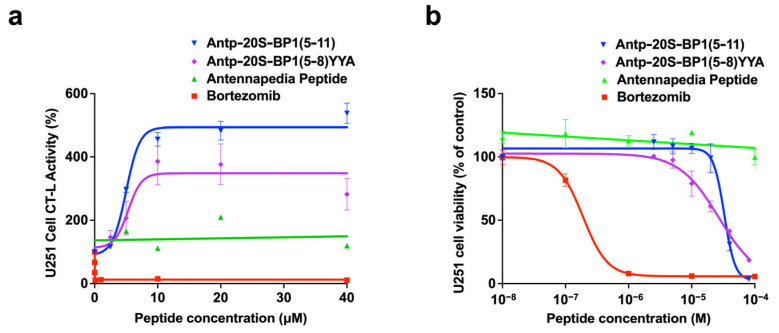
Profiles of the effects of Antp-20S-BP1(5-11) and Antp-20SP-BP1(5-8)YYA on U251 cells. (**a**) Inhibition of the CT-L activity of intracellular proteasomes by peptides verified for their action on U251 cells. Means ± SD, *n* = 3. (**b**) Survival curve of U251 cells after peptide treatment. Antp-20S-BP1(5-11) (blue), Antp-20S-BP1(5-8)YYA (purple), antennapedia peptide (green), and bortezomib (red). Means ± SD, *n* = 3.

The antennapedia peptide (Antp) is a cell-penetrating peptide (CPP) consisting of 16 amino acids [27]. To examine the effect of 20S-BP1(5-11) and 20S-BP1(5-8)YYA on intracellular proteasomes, Antp-20S-BP1(5-11) and Antp-20S-BP1(5-8)YYA with the Antp sequence attached to their N-termini were synthesized. They showed 20S CP inhibition in vitro (Figure S4 and Table 1). When each synthetic peptide was added to the culture medium of human glioblastoma U251 cells, and the activity of intracellular proteasomes was measured, the Antp sequence alone did not affect the CT-L activity of intracellular proteasomes, whereas Antp-20S-BP1(5-11) and Antp-20S-BP1(5-8)YYA promoted CT-L activity by 4.6-fold and 3.8-fold, respectively, at 10 μM, although bortezomib as a control proteasome inhibitor did not increase CT-L activity to the same degree (Figure 6a). These results suggest that these synthetic peptides enhanced intracellular 20S CP activity. Based on the in vitro activation results for 20S CP, we expected that Antp-20S-BP1(5-11) would show weaker activation than Antp-20S-BP1(5-8)YYA, but their degree of activation was almost the same. This may be due to the presence of the added CPP sequence or to differences in peptide action between in vitro and in vivo experiments. Antp-20S-BP1(5-11) also increased intracellular 20S CP activity in other human cell lines, RPMI8226 and SH-SY5Y, although their profiles were somewhat different (Figure S5). Furthermore, Antp-20S-BP1(5-11) and Antp-20S-BP1(5-8)YYA induced cell death in U251 cells with a 50% inhibition of cell proliferation (Cyt_50_) value of 33.5 μM and 26.9 μM, respectively, and bortezomib also induced cancer cell death (Figure 6b). Since an equilibrium exists between the 20S CP and the 26S holoenzyme, the activation of 20S CP may indirectly interfere with ubiquitin-dependent protein degradation [16]. We suggest that the peptide enhances 20S CP activity and affects the homeostasis of proteins, including those involved in the proliferation of cancer cells, leading to the inhibition of their growth inhibition.

### 2.7. Enhanced Degradation of 20S CP Substrate Proteins Harboring a 20S-BP1 Sequence

**Figure 7:**
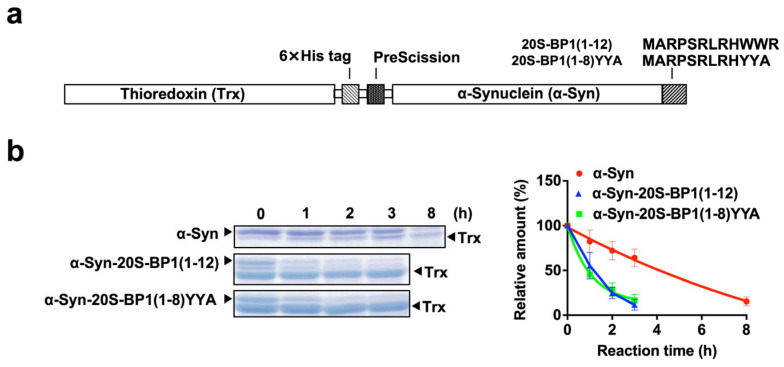
Fusion of 20S-BP1(1-12) or 20S-BP1(5-8)YYA to α-synuclein (α-Syn) increases its degradation by 20S CP. (**a**) Schematic diagram of thioredoxin (Trx)-α-Syn fused to 20S-BP1(1-12) or 20S-BP1(5-8)YYA. (**b**) α-Syn degradation over time. Coomassie brilliant blue staining of SDS-PAGE gels of α-Syn proteins treated with 20S CP digestion (left). ImageJ quantitation of band intensity of α-Syn without (red) and with 20S-BP1(1-12) (blue) or 20S-BP1(5-8)YYA (green) (right). Means ± SD, *n* = 3.

Alpha-synuclein (α-Syn) is known to be degraded slowly by 20S CP alone [30,31]. The structure–activity relationships described above suggest that 20S-BP1(5-11) is the shortest amino acid sequence with binding affinity for the 20S CP, despite the strong stimulation of the 20S CP by 20S-BP1(1-12), indicating that the presence of Met^1^, Arg^3^, and Arg^12^ may positively affect 20S CP activation (Table 1). The amino acid sequence of 20S-BP1(1-12) (Met-Ala-Arg-Pro-Ser-Arg-Leu-Arg-His-Trp-Trp-Arg) was added to the C-termini of α-Syn to examine changes in its susceptibility to degradation. Thioredoxin (Trx) fusion proteins of α-Syn, with and without 20S-BP1(1-12) or 20S-BP1(1-8)YYA, were prepared (Figure 7a and Supplementary Information Figures S6–S8). These were then added to purified 20S CP and their half-lives were assessed by SDS-PAGE (Figure 7b). The half-life of α-Syn fused to Trx was 4.0 h, whereas the half-life of α-Syn fused to 20S-BP1(1-12) or 20S-BP1(1-8)YYA was 0.9 h. The further digestion of Trx-cleaved α-Syn with the 20S CP degraded only α-Syn, to which the 20S-BP1(1-12) or 20S-BP1(1-8)YYA sequence had been added, but not Trx generated by prior cleavage. These results indicate that when the 20S-BP1(1-12) or 20S-BP1(1-8)YYA is fused to the substrate protein, degradation by the 20S CP is accelerated.

**Figure 8:**
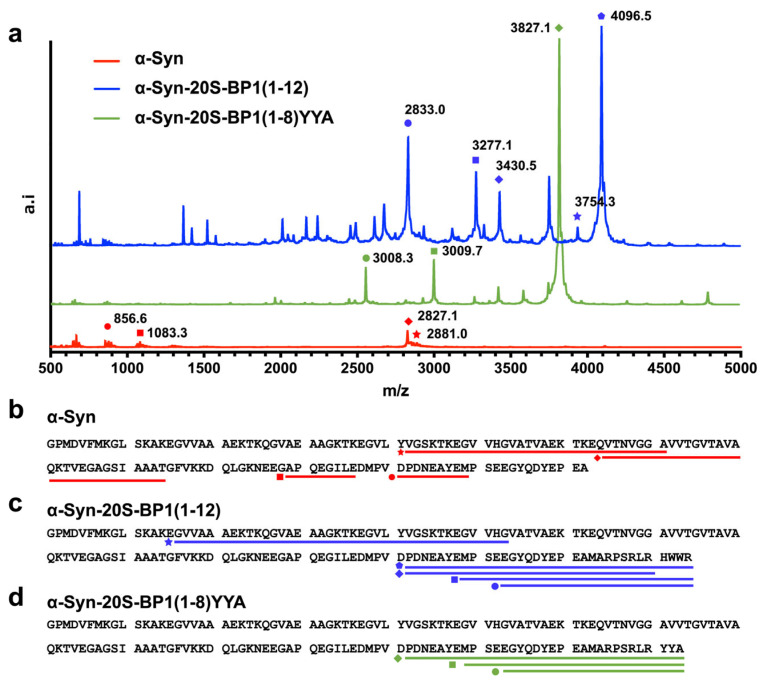
(**a**) Mass spectra of the peptides released by 20S CP digestion of recombinant α-Syn proteins. Each colored spectrum was obtained from digests of recombinant α-Syn (red), α-Syn-20S-BP1(1-12) (blue), or α-Syn-20S-BP1(1-8)YYA (green). (**b**–**d**) Graphical representation of the digestion map generated from the degradation of recombinant α-Syn protein by the 20S CP. Red, blue, and green bars below the protein sequences indicate the peptides released by 20S CP digestion and detected by MALDI-TOF-MS. The color and the shape of the dots indicate the peptide fragments corresponding to the detected peaks in the mass spectra.

The molecular mass distributions of the peptides degraded by 20S CP were determined using MALDI-TOF MS. α-Syn (fused to Trx) was completely cleaved and only a few peptide fragments with a molecular weight above *m*/*z* 500 were detected, whereas the cleavage of α-Syn fused to 20S-BP1(1-12) or 20S-BP1(1-8)YYA resulted in the generation of relatively large peptide fragments (*m*/*z* > 2500). The *m*/*z* of these fragments were in complete agreement with the predicted molecular masses of the C-terminal amino acid sequences (122–142) of α-Syn fused to 20S-BP1(1-12) or 20S-BP1(1-8)YYA (Table 2), indicating that these regions of α-Syn are not easily degraded by the 20S CP. Taken together, these results suggest that the region containing either of these peptide sequences induced substrate degradation through its interaction with the α-ring of the 20S CP, but did not reach the internal active site of the 20S CP because the C-terminus was fixed at the K-pocket position. Consistent with this hypothesis, if the amino acid sequence linking the C-terminus of α-Syn to 20S-BP1 is fixed in the K-pocket of the α1 subunit and extended through the gate toward the β-annulus, the cleavage site of the peptide would be located close to the active site (Figure S9). This position is 12–13 nm from the K-pocket on the α-ring. After cleavage, if the stretched peptide moves in the opposite direction and exits the gate, long peptide fragments would result in α-Syn-20S-BP1(1-12) and α-Syn-20S-BP1(1-8)YYA protein digests (Figure 8).

### 2.8. Docking Study of the 20S-BP1 on the α-Ring of the 20S CP

**Figure 9:**
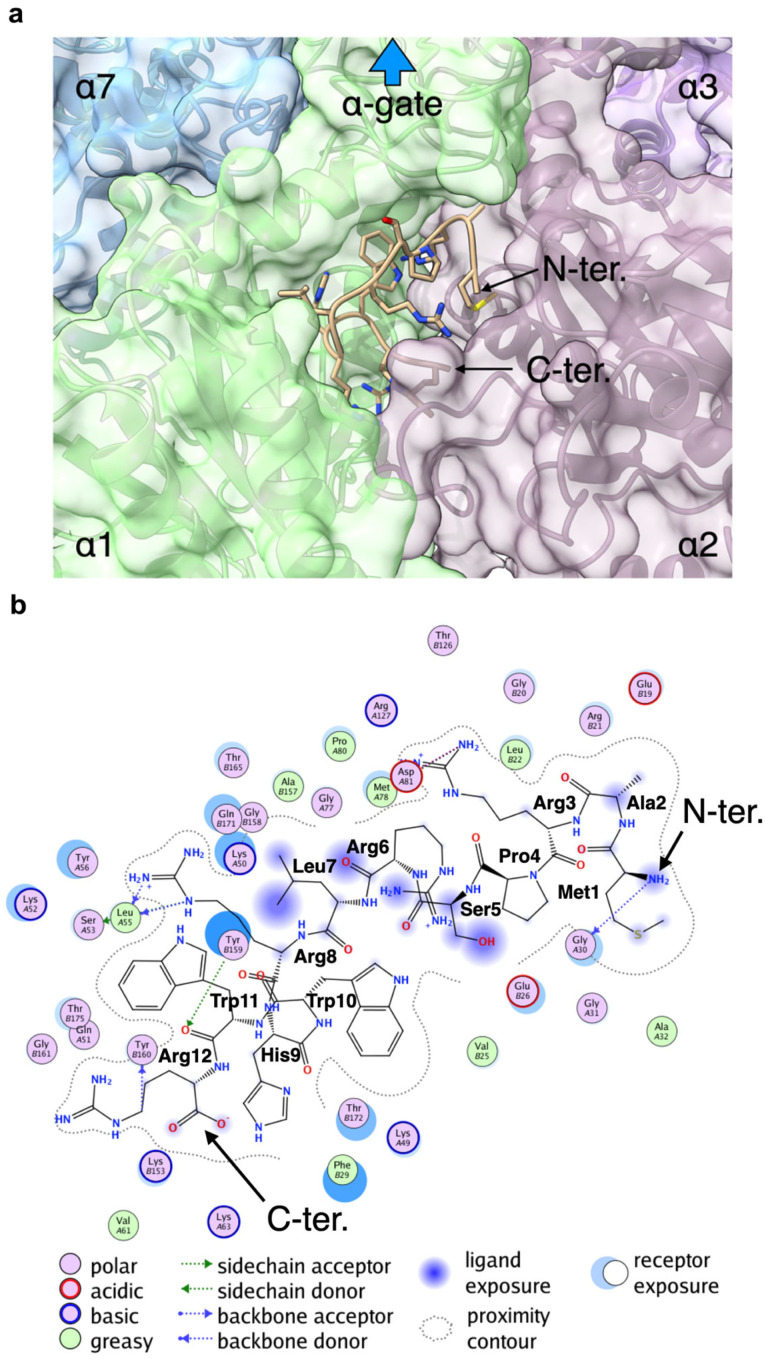
(**a**) The best model structure of the docking results. The backbone structure of 20S-BP1(1-12) is shown in the cartoon model, and the side chains are shown in the stick model. The subunits (α1, α2, α3, and α7) of the α-ring are shown in different colors in both the cartoon and surface models. This figure was created using ChimeraX version 1.16 [35]. (**b**) The interaction diagram created by MOE. The chain identifiers A and B in front of the residue number in the circle indicate α2 and α1, respectively.

We next simulated the docking of 20S-BP1(1-12) to the α-ring of the 20S CP. In the previous experiments (Figure 7 and Figure 8, Table 2), MALDI-TOF MS showed that the attachment of 20S-BP1(1-12) at the C-terminus of α-Syn degraded itself rapidly, suggesting that the peptide sequences interacted with the α-ring of the 20S CP and induced substrate degradation. Although the binding site of 20S-BP1(1-12) is assumed to be the K-pocket on the α-ring, the actual binding site is unknown. Therefore, we employed global peptide docking to search for binding sites on the α-ring and docking poses of 20S-BP1(1-12) by using CABS-dock standalone [32]. From the top 10 docking poses generated by CABS-dock standalone, we selected possible docking poses where 20S-BP1(1-12) interacted with the α-ring of the 20S CP and reconstructed the all-atom models of these using MODELLER [33]. As a result, eight docking poses were excluded because they interacted with the β-ring interface or overlapped with the α-ring. The DOPE scores of the all-atom models of the remaining two docking poses were −181,688.75 and −162,862.28 (zDOPE: −0.14068 and 0.65580), respectively. In the best all-atom model (model 1), 20S-BP1(1-12) docked with the K-pocket formed by α1 and α2 subunits (Figure 9a). In the other model (model 2), 20S-BP1(1-12) docked with the pocket formed by α4 and α5 subunits (Figure S11a). To assess the stability of these models, we conducted a 50 ns MD simulation for each model using GROMACS [34]. Although the RMSD value of 20S-BP1(1-12) increased during the first part of the simulation for model 1, it did not increase further after about five ns (Figure S10a). During the simulation for model 1, the RMSD values of 20S-BP1(1-12) were less than 2.0 Å and were not appreciably different from those of the α-ring subunits (Figure S10b). The RMSF values of the α carbon atoms in 20S-BP1(1-12) were less than 1.0 (Figure S10c), and were comparable to those in the α-ring subunits (Figure S10d). On the other hand, the values of RMSD and RMSF of model 2 were higher than those of model 1 (Figure S11b–e). These results indicate that 20S-BP1(1-12) binds more stably to the K-pocket formed by α1 and α2 subunits than to the pocket formed by the α4 and α5 subunits. The interaction calculation for model 1 showed that amino acid residues Met^1^, Ala^2^, Ser^5^, Arg^6^, and Leu^7^ tended to be exposed to the solvent, while the amino acid residues Arg^8^, His^9^, Trp^10^, Trp^11^, and Arg^12^ tended to interact with residues located at the bottom of the K-pocket (Figure 9b). To assess the stability of the model, we conducted a 50 ns MD simulation using GROMACS [34]. Although the RMSD value of 20S-BP1(1-12) increased during the first part of the simulation, it did not increase further after about five ns (Figure S10a). The RMSD values of 20S-BP1(1-12) during the simulation were less than 2.0 Å and were not appreciably different from those of the α-ring subunits (Figure S10b). The RMSF values of the α carbon atoms in 20S-BP1(1-12) were less than 1.0 (Figure S10c), and were comparable to those in the α-ring subunits (Figure S10d). These results indicate that 20S-BP1(1-12) can stably bind to the K-pocket. Although the direction of the N-terminus of 20S-BP1(1-12) in the model was the opposite of that in the α-gate (Figure 9a), the N-terminus was exposed to the solvent (Figure 9b). In addition, the fluctuations of Met^1^ and Ala^2^ were 0.723 and 0.756, respectively, and these values were the highest in 20S-BP1(1-12), except for that of Arg^6^ (0.855). Therefore, when α-Syn-20S-BP1(1-12) binds to the K-pocket via the fused 20S-BP1(1-12) peptide, α-Syn can be directed to and inserted into the α-gate.

### 2.9. Final Considerations

The 20S CP has been suggested to act directly on intrinsically unstructured or oxidized proteins and to serve as an independent protease [36,37]. Since there are many interaction sites on the surface of the α-ring for loosely folded polypeptides, some regions of substrate proteins are postulated to bind to these sites and facilitate the gating of the α-ring, and interactions with specific α-subunits have been reported for several substrate proteins [23,29]. For example, α-Syn, which is sensitive to degradation by the stand-alone 20S CP, interacts with the 20S CP via its N-terminal region. Assuming that the 20S CP selects specific substrates, the area around the K-pocket on the upper surface of the α-ring may recognize a particular amino acid sequence in α-Syn. This amino acid sequence in 20S CP substrates may have the features of a consensus sequence intrinsic to 20S CP substrate proteins that opens the α-gate and leads to substrate entry into the 20S CP.

Degrons are generally defined as structural motifs in polyubiquitinated proteins, which are substrates of the 26S proteasome, and are recognized by E3 ubiquitin ligase [30]. As sites that regulate protein levels, degrons play an important role in a variety of cellular processes, including cell cycle progression and the monitoring of cellular hypoxia. In recent years, accumulating evidence indicates that the 20S CP alone can degrade proteins in a ubiquitin-independent manner, and internal sequences of substrate proteins may act as 20S CP-specific degrons (i.e., sequences that induce degradation and promote degradation by the 20S CP). It has been reported that PEST sequences in 20S CP substrate proteins, which are rich in proline (P), glutamate (E), serine (S), and threonine (T), interact with the 20S CP via structural destabilization during degradation and that these PEST sequences then function as 20S CP-specific degrons for the promotion of degradation by the 20S CP [31,38]. Although the 20S CP interacting peptides obtained from the random sequence pool in this study did not have the characteristics of PEST sequences [39], they may function as the interacting amino acid sequences of native substrates of the 20S CP-like degrons. One reason for this is that many of the exogenous peptides that promote 20S CP activation are those rich in basic amino acids [18,19,20]. The 20S-BP1(1-12) used in this study is also enriched in basic amino acids, and some Arg residues either do not affect (Arg^12^) or attenuate (Arg^3^) the inhibitory activity of this peptide in the presence of SDS, but positively increase the activity of 20S CP in the absence of SDS. The reason for the opposing effects of this peptide is not yet fully understood, and a structural biology approach using cryo-EM and other methods will be needed to solve this issue.

The amino acid sequence of 20S-BP1(1-12) may contain the consensus sequence of a putative substrate protein of the 20S CP. To determine whether the position of this sequence in a protein affected its activity, we inserted it at the C-terminus of α-Syn. The results showed that the sequence increased the degradation of α-Syn. However, this was a first attempt, and it is possible that if the sequence is shortened or lengthened, the degradation efficiency and cleavage position of the substrate protein by the 20S CP would change. It is also important to determine the extent to which substrate degradation is enhanced when the 20S-BP1 sequence is inserted into a region other than the C-terminus of α-Syn. This study is currently underway, and the results could make the cleavage model shown in Figure S9 more robust.

Recent studies have focused on compounds that induce specific protein degradation by targeting ubiquitin ligases, such as PROTAC, as a recent therapeutic modality that targets disease-causing proteins [40]. These compounds promote protein degradation via the 26S holoenzyme. However, the 20S CP, which is presumably responsible for the independent intracellular proteolytic system, has been shown to play an important role in cellular tolerance to oxidative and hypoxic stress [16]. Studying the mechanism of selective activation of the 20S CP is important for a number of reasons. First, since the 20S CP plays an important role in stressed cells, targeting the 20S CP may help to elucidate new pathological mechanisms and develop novel therapies. In addition, since the mechanism of action is different from that of existing therapeutic agents targeting the 26S proteasome, it may help develop therapeutic agents with fewer side effects and less resistance. Furthermore, since the activation of the 20S CP is directly related to cell survival, it could provide new options for the treatment of cancer and neurodegenerative diseases. Thus, the study of 20S CP activation has great potential for increasing understanding of new disease mechanisms and for drug discovery [41]. The development of compounds that exploit this mechanism may open new avenues for the treatment of various diseases.

## 3. Materials and Methods

All reagents for biochemical experiments were obtained from FUJIFILM Wako Pure Chemical Co. (Osaka, Japan). Synthetic oligonucleotides were synthesized by Eurofins Genomics K.K. (Tokyo, Japan). *N*-hydroxysuccinimide ester (NHS)-activated FG beads were purchased from Tamagawa Seiki Co., Ltd. (Nagano, Japan). In vitro RNA transcription was performed using a T7 RiboMAX Express Large-Scale RNA Production System (Promega Co., Madison, WI, USA) and purified using an RNeasy Mini Kit (Qiagen N.V., Hilden, Germany). DNA was purified using a FavorPrep PCR Clean-Up Mini Kit (Favorgen, Ping Tung, Taiwan). Electrophoresis analyses of DNA and RNA were performed at 60 °C using 8 M urea for 4% denaturing PAGE and 0.5× Tris/Borate/EDTA (TBE) as the running buffer. SDS-PAGE was performed at room temperature using Tris-HCl and gels with or without urea, followed by CBB staining, silver staining, and Western blotting, depending on the application. All DNA and RNA samples were visualized using SYBRGold Nucleic Acid Gel Stain (Thermo Fisher Scientific Inc., Waltham, CA, USA).

### 3.1. The cDNA Display Method

Candidate peptides interacting with the 20S CP were obtained using a cDNA display method, as described previously [25]. In this study, we used a cDNA display library that encoded random amino acid sequences of eight residues. The 20S CP was immobilized on NHS-activated FG beads (Tamagawa Seiki Co., Ltd.) using amine coupling chemicals. The cDNA display library was diluted in 100 µL of selection buffer (phosphate-buffered saline with Tween-20 (PBST) (pH 7.4) containing 0.1 M NaCl) and incubated with 10 µL of 20S CP-immobilized beads (200 μg) containing ~10 pmol immobilized 20S CP (based on CT-L activity). Beads were washed with 200 µL of washing buffer (0.25 M NaCl in PBST, pH 7.4). After washing, beads were suspended in 35 µL of elution buffer (0.1 M KOH) and incubated at 25 °C for 10 min, and the supernatant was collected (Sup). This was immediately neutralized with 15 µL of 1 M Tris-HCl (pH 7.5). The cDNA portion of the purified sample was amplified by PCR for the next round of selection.

Next-generation sequencing (NGS) analysis was performed using a MiSeq System (Illumina Inc., San Diego, CA, USA) on PCR products from cycles 0–5 for the cDNA display libraries. A total of 15 Gb of data was collected, and the average read length was 190 bp. Sickle (ver.1.33) was used for quality filtering [42], Fastxtoolkit (ver.0.0.13.2) was employed for sequence trimming [43], and FLASH (ver.1.2.10) was used to facilitate pair-end binding [44]. The data obtained were subjected to data analysis using Fastaptmer ver.1.0.3 [45].

### 3.2. Peptide Synthesis

Peptides were synthesized by standard Fmoc/HBTU chemistry. Crude peptides were purified with a high-performance liquid chromatography (HPLC) system (Gilson Inc., Middleton, WI, USA) equipped with a COSMOSIL 5C18-AR-II reversed-phase column (4.6 × 150 mm; Nacalai Tesque Inc., Kyoto, Japan). Purity was confirmed with a Spiral-TOF JMS-S3000 mass spectrometer (JEOL Ltd., Kyoto, Japan).

### 3.3. Proteasome Fluorometric Substrate Assay

CT-L, PGPH, and T-L proteasome activities were determined by measuring the degradation rates of the fluorometric substrates succinyl-LLVY-AMC, Z-LLE-AMC, and Boc-LRR-AMC, respectively (Peptide Institute Inc., Osaka, Japan) [23]. Purified human 20S proteasome (100 ng; Enzo Life Sciences Inc., Farmingdale, NY, USA) was incubated with 50 mM (CT-L) or 20 mM (PGPH and T-L) fluorometric peptide substrate in the presence of varying concentrations of inhibitory compounds in 100 μL of assay buffer comprising 25 mM 2-[4-(2-hydroxyethyl)-1-piperazinyl]ethanesulfonic acid (HEPES) and 0.5 mM ethylenediaminetetraacetic acid (EDTA) with or without 0.03% SDS at 37 °C. Reactions were monitored by measuring 7-amino-4-methylcoumarin (AMC) product formation (λ_ex_ 380 nm, λ_em_ 460 nm) for 1 h. The half maximal inhibitory concentration (IC_50_) or maximum activating concentration (AC_max_) was determined for each compound from the respective proteasome activity curves.

### 3.4. Fluorescent Microscale Thermophoresis (MST) Assay

The dissociation constant (K_D_) values of peptides for the 20 CP were determined using an MST instrument (2Bind GmbH, Regensburg, Germany) using the control software, MO.Control version1.6.1. For protein labeling, human 20S CP was labeled using a RED-NHS 2nd Generation Protein Labeling Kit (MO-L011; NanoTemper Technologies GmbH, München, Germany), according to the manufacturer’s instructions, supplied with the labeling buffer. After labeling, protein was eluted into 20 mM Tris-HCl (pH 8.0), 50 mM NaCl, 1 mM EDTA, and 1 mM tris(2-carboxyethyl)phosphine. MST binding experiments were conducted in binding buffer comprising 50 mM Tris-HCl pH 8.0, 100 mM KCl, 1 mM EDTA, 0.05% Tween-20, 5 nM 20S CP, 9 μM Bortezomib, 3.12% glycerol, and 1.25% dimethylsulfoxide (DMSO). The K_D_ of the interaction was determined by fitting the data using an MST standard fit algorithm (MO. Affinity Analysis version 2.3) derived from the law of mass action. To calculate the fraction bound, the DFnorm value of each point was divided by the amplitude of the fitted curve, resulting in values from 0 to 1 (0 = unbound, 1 = bound). Error bars represent the standard deviation (SD) of three or four independent experiments.

### 3.5. Photoaffinity Labeling

A mixture of 50 nmol peptide and 50 nmol sulfo-N-hydroxysuccinimidyl-2-(6-[biotinamido]-2-(p-azido benzamido)-hexanoamido) ethyl-1,3′-dithioproprionate (Sulfo-SBED, Thermo Fisher Scientific Inc.) was added to 10 µL of *N,N*-dimethylformamide containing 1% triethylamine for 48 h to synthesize the photoreactive biotinylated peptide BAED-20S-BP1(5-12). After the reaction, the product was purified by reversed-phase HPLC, and molecular weight was confirmed by mass spectrometry, as described above.

Photoaffinity labeling of the human 20S proteasome (10 ng) was performed using 10 µM BAED-20S-BP1(5-12) in 10 µL of reaction buffer (25 mM HEPES, 0.5 mM EDTA, 0.03% SDS, pH 8.0) for 30 min in the dark. The reaction mixture was exposed to UV light (365 nm) for 30 min on ice, and proteins were separated by SDS-PAGE, transferred onto a polyvinylidene difluoride membrane (ATTO Co., Ltd., Tokyo, Japan), and probed with horseradish peroxidase-conjugated streptavidin (Cytiva, Danaher Co., Washington, DC, USA). Chemiluminescence images were obtained using an ImageQuant LAS-4000 instrument (Cytiva). Isoelectric focusing for 2D-PAGE was performed using a DiscRun AE-6541 apparatus (ATTO Co., Ltd.) according to the manufacturer’s protocol. To identify the subunits of the 20S CP by LC-MS analysis, all bands visible following CBB staining were excised, dehydrated with acetonitrile for 10 min, and centrifuged in vacuo to dryness. Dried samples were placed in trypsin solution, and hydrolysis was carried out at 37 °C for 16 h. Reaction products were recovered using 0.1% formic acid and analyzed by LC-MS-IT-TOF (Shimadzu Corporation, Kyoto, Japan) and Mascot Version 2.4 (Matrix Science, London, UK).

### 3.6. Cell Growth Inhibition Assay

The U251 cell line was kindly provided by Prof. Koji Kawakami (Kyoto University). RPMI8226 and SH-SY5Y cell lines were obtained from RIKEN BRC Cell Bank (Chiba, Japan). U251 and RPMI8226 cells were grown and maintained in RPMI1640 medium (Nacalai Tesque Inc.) supplemented with 10% fetal bovine serum, 100 units/mL penicillin, and 100 μg/mL streptomycin at 37 °C in 5% CO_2_. SH-SY5Y cells were grown and maintained in DMEM medium (Nacalai Tesque Inc.) supplemented with 10% fetal bovine serum, 100 units/mL penicillin, and 100 μg/mL streptomycin at 37 °C in 5% CO_2_.

U251, RPMI8226, or SH-SY5Y cells were seeded in triplicate wells in a 96-well plate at a density of 5 × 10^3^ cells/well and incubated with various concentrations (0.1–100 µM) of inhibitory compounds dissolved in DMSO for 24 or 48 h. The amount of intracellular ATP was determined by CellTiter-Glo Luminescent Cell Viability Assay (Promega Co.) according to the manufacturer’s protocol. Intracellular proteasome activity was measured using the Proteasome-Glo Assay reagent (Promega Co.) according to the manufacturer’s protocol [46].

### 3.7. The 20S Proteasome Assay of Substrate Proteins

α-Syn was expressed as a thioredoxin (Trx)-fused protein in *Escherichia coli* cells carrying the corresponding synthetic gene in the pET32a vector (Novagen, Merck KGaA, Darmstadt, Germany), purified on Ni chelate columns, and incubated with human 20S proteasomes. The Trx region was released by digestion using PreScisson protease (Cytiva). The 20S proteasome ratio was 1:100 pmol and 1:10 pmol for α-Syn. Degradation experiments were carried out at 37 °C in 20 mM HEPES pH 7.4 in a 10 μL total sample volume for 1 h. The reaction was stopped with 4× SDS sample buffer; samples were boiled for 5 min at 95 °C, loaded (8 μL) onto a 12% gel, and separated by SDS-PAGE; and protein bands were detected by CBB staining.

### 3.8. Docking Study of 20S-BP1(1-12) and α-Ring of 20S CP

The structural data of human 20S CP (PDB ID: 5le5) [47], obtained at a higher resolution, were chosen and chains A to G, which make up the α-ring, were extracted. Because the binding site of 20S-BP1(1-12) on the α-ring is unknown, we employed CABS-dock standalone [32] for global peptide docking, which searches for binding sites and docking poses. The CABS-dock standalone program was executed using the predicted secondary structure information (CCCHHHHHHHHC) of 20S-BP1(1-12) from PSSpred [48]. The top 10 docking poses generated by the CABS-dock standalone were evaluated, and any poses where 20S-BP1(1-12) interacted with the β-ring interface or overlapped with the α-ring were excluded. As the docking pose generated by the CABS-dock standalone consists of only α carbon atoms, we reconstructed five all-atom models using MODELLER [33] version 10.4 and chose the best all-atom model based on its DOPE score for each remaining pose. To calculate interactions between 20S-BP1(1-12) and the α-ring, we used the “Ligand Interactions” function following the execution of “Protonate 3D” with default parameters in Molecular Operating Environment (MOE) version 2022.02 (Chemical Computing Group ULC, Montreal, QC, Canada).

All-atom molecular dynamics (MD) simulation was performed to assess the stability of the docking pose. GROMACS [34] version 2023 with the CHARMM27 force field was used for potential-energy minimization (EM) and MD simulations. The system contained the docking pose, 112,116 water molecules (SPC/E model), and 15 Na^+^ ions in the cubic box. The docking pose was positioned at a minimum distance of 10 Å from the box edge. After 50,000-step EM, 150 ps NVT MD and 150 ps NPT MD were performed with a 1 fs MD time step to equilibrate the system, after which a 50 ns NPT MD was conducted using a 2 fs MD time step for the MD production. The target temperature and pressure were set to 300 K and 1.0 atm, respectively. The electrostatic interactions were calculated using the PME algorithm. Coordinates were saved every 100 ps in the production MD. From the trajectories obtained from the MD simulation, the root mean square deviation (RMSD) and root mean square fluctuation (RMSF) of α carbon atoms were calculated for 20S-BP1(1-12) and each subunit of the α-ring.

## 4. Conclusions

In this study, an in vitro synthetic molecular evolution approach, specifically a cDNA display, was used to generate peptides that bind to the 20S core particle (CP). These peptides effectively inhibited the proteolytic activity of SDS-activated 20S CP. Remarkably, modifying the C-terminal of these peptides with the HbYX motif (Tyr-Tyr-Ala) increased the proteolytic function of the latent 20S CP. Photoaffinity labeling analysis revealed that this peptide directly interacts with the α-ring of the 20S CP. Since the α-ring contains a site called the K-pocket that interacts with the HbYX motif and is involved in controlling gate opening, it was inferred that this artificially generated peptide sequence functions to open the substrate uptake gate of 20S CP by acting on the K-pocket [9,49]. Additionally, these 20S CP-binding peptides, when fused with a cell-penetrating sequence, increased the intracellular proteasome activity, ultimately leading to cell apoptosis. Furthermore, the attachment of the peptide sequence to α-Syn promoted its degradation by the 20S CP in vitro. Several known substrates of the free 20S CP are assumed to have interaction sites that open an α-gate, allowing them entry into the 20S chamber [9]. Our cDNA display technique identified many amino acid sequences that potentially act on the 20S CP. Notably, among the peptides examined, 20S-BP1(1-12) (Met-Ala-Arg-Pro-Ser-Arg-Leu-Arg-His-Trp-Trp-Arg) resulted in the strongest activation of the 20S CP. However, when the N-terminal Met residue and C-terminal Arg were removed, activation was largely lost, resulting in inhibition only in the presence of SDS. The analysis of the structure–activity relationships of these peptides may reveal a consensus sequence for the regulation of the 20S CP, and further work is underway to identify this sequence.

## Figures and Tables

**Table 1:** Effects of each peptide on 20S proteasome CT-L activity.

Name	Sequence	SDS (+)	SDS (−)	
		IC_50_ ± SD (μM)	Maximum Active Concentration (μM)	Activity Increase (%)
20S-BP1(1-17)	MARPSRLRHWWRLRRRV	1.35 ± 0.21	-	-
20S-BP1(1-12)	MARPSRLRHWWR	0.93 ± 0.30	10	1215
20S-BP1(6-17)	RLRHWWRLRRRV	>10	-	-
20S-BP1(1-11)	MARPSRLRHWW	0.84 (±0.03)	0.7	149
20S-BP1(1-10)	MARPSRLRHW	2.31 (±0.41)	6.7	197
20S-BP1(1-9)	MARPSRLRH	7.46 (±1.11)	66.7	310
20S-BP1(1-8)	MARPSRLR	8.51 (±0.25)	66.7	237
20S-BP1(1-7)	MARPSRL	>50	66.7	179
20S-BP1(1-6)	MARPSR	>50	0.7	107
20S-BP1(2-12)	ARPSRLRHWWR	4.26 (±1.76)	0.7	172
20S-BP1(3-12)	RPSRLRHWWR	>50	0.7	154
20S-BP1(4-12)	PSRLRHWWR	0.83 (±0.11)	2.0	163
20S-BP1(5-12)	SRLRHWWR	0.82 (±0.11)	2.0	207
20S-BP1(6-12)	RLRHWWR	1.64 (±0.20)	2.0	168
20S-BP1(7-12)	LRHWWR	1.34 (±0.11)	6.7	157
[Ala^1^]20S-BP1(1-12)	AARPSRLRHWWR	3.91 (±0.30)	-	-
[Ala^3^]20S-BP1(1-12)	MAAPSRLRHWWR	1.10 (±0.01)	-	-
[Ala^4^]20S-BP1(1-12)	MARASRLRHWWR	3.02 (±0.06)	-	-
[Ala^5^]20S-BP1(1-12)	MARPARLRHWWR	5.46 (±0.14)	-	-
[Ala^6^]20S-BP1(1-12)	MARPSALRHWWR	1.59 (±0.06)	-	-
[Ala^7^]20S-BP1(1-12)	MARPSRARHWWR	5.09 (±0.13)	-	-
[Ala^8^]20S-BP1(1-12)	MARPSRLAHWWR	1.00 (±0.08)	-	-
[Ala^9^]20S-BP1(1-12)	MARPSRLRAWWR	2.53 (±0.07)	-	-
[Ala^10^]20S-BP1(1-12)	MARPSRLRHAWR	2.54 (±0.19)	-	-
[Ala^11^]20S-BP1(1-12)	MARPSRLRHWAR	2.30 (±0.07)	-	-
[Ala^12^]20S-BP1(1-12)	MARPSRLRHWWA	0.72 (±0.02)	-	-
20S-BP1(1-8)TYN	MARPSRLRTYN	9.78 (±1.51)	30.0	260
20S-BP1(1-8)LYL	MARPSRLRLYL	2.51 (±0.18)	1.0	183
20S-BP1(1-8)FYK	MARPSRLRFYK	1.26 (±0.13)	1.0	231
20S-BP1(1-8)KPV	MARPSRLRKPV	14.7 (±0.56)	3.0	313
20S-BP1(1-8)LWK	MARPSRLRLWK	2.55 (±0.16)	1.0	250
20S-BP1(1-8)YYA	MARPSRLRYYA	3.80 (±0.81)	10.0	467
20S-BP1(2-8)YYA	ARPSRLRYYA	1.81 (±0.14)	10.0	334
20S-BP1(3-8)YYA	RPSRLRYYA	1.59 (±0.00)	10.0	513
20S-BP1(4-8)YYA	PSRLRYYA	9.92 (±0.25)	53.0	668
20S-BP1(5-8)YYA	SRLRYYA	14.7 (±0.83)	100.0	481
20S-BP1(6-8)YYA	RLRYYA	4.44 (±0.23)	100.0	644
20S-BP1(7-8)YYA	LRYYA	>50	N.D.	N.D.
20S-BP1(1-8)YY	MARPSRLRYY	2.64 (±0.28)	1.2	204
20S-BP1(1-8)Y	MARPSRLRY	3.71 (±0.80)	N.D.	N.D.
Rpt2-C	QEGTPEGLYL	>50	N.D.	N.D.
Rpt5-C	KKKANLQYYA	28.1 (±3.29)	100.0	1046
Rpt3-C	KDEQEHEFYK	>50	N.D.	N.D.
AntP-20S-BP1(5-11)	RQIKWFQNRRMKWKKSRLRHWW	1.34 (±0.11)	-	-
AntP-20S-BP1(5-8)YYA	RQIKWFQNRRMKWKKSRLRYYA	0.79 (±0.05)	-	-
Antennapedia peptide	RQIKWFQNRRMKWKK	>50	-	-

Control activity (100%) was defined as the released fluorophore after a 60 min reaction with 20S CP (100 μg) and Suc-LLVY-AMC (10 nmol). Data were collected in triplicate, and results are shown with standard deviation. N.D.: not detected. -: not determined.

**Table 2:** MALDI-TOF MS Results.

Name	Range	Target Mass (*m*/*z*)	Measured Mass (*m*/*z*)
α-Syn	42–71	2881.5	2881.0
	64–94	2826.5	2827.1
	121–129	1082.4	1083.3
	106–116	856.4	856.6
α-Syn-20S-BP1(1-12)	122–142 + 20S-BP1(1-12)	4095.8	4096.5
	16–53	3754.0	3754.3
	122–142 + 20S-BP1(1-8)	3430.5	3430.5
	129–142 + 20S-BP1(1-12)	3277.5	3277.1
	133–142 + BP1(1-12)	2833.3	2833.0
α-Syn-20S-BP1(1-8)YYA	122–142 + 20S-BP1(1-8)YYA	3826.6	3827.1
	129–142 + 20S-BP1(1-8)YYA	3008.3	3009.7

## Data Availability

The datasets used and analyzed during the current study are available from the corresponding author upon reasonable request.

## Supplementary Materials

The supplementary information can be downloaded at: https://www.mdpi.com/article/10.3390/ijms242417486/s1.
