# Supplementary material for: Acceleration of Protein Degradation by 20S Proteasome-Binding Peptides Generated by In Vitro Artificial Evolution
Source: Int J Mol Sci. 2023 Dec 14;24(24):17486. doi: 10.3390/ijms242417486 (PMC10743564; doi:10.3390/ijms242417486)
Supplement: Supplementary file 1 [file ijms-24-17486-s001.zip › ijms-2712888-supplementary.pdf]

Supplementary Information

# Acceleration of Protein Degradation by 20S Proteasome-binding Peptides Generated by In Vitro Artificial Evolution

*Yunhao Zhu [1], Kaishin Shigeyoshi [1], Yumiko Hayakawa [1], Sae Fujiwara [1], Masamichi Kishida [2], Hitoshi Ohki [2], Tomohisa Horibe [1], Masafumi Shionyu [1], Tamio Mizukami [1,3] and Makoto Hasegawa [1,*]*

[1] Graduate School of Bioscience, Nagahama Institute of Bio-Science and Technology, 1266 Tamura-cho, Nagahama 526-0829, Japan

[2] Modality Research Laboratories, Biologics Division, Daiichi Sankyo Co., Ltd., 1-2-58, Hiromachi, Shinagawa-ku, Tokyo 140-8710, Japan

[3] Frontier Pharma Inc., 1281-8 Tamura, Nagahama 526-0829, Japan

*Correspondence: m_hasegawa@nagahama-i-bio.ac.jp

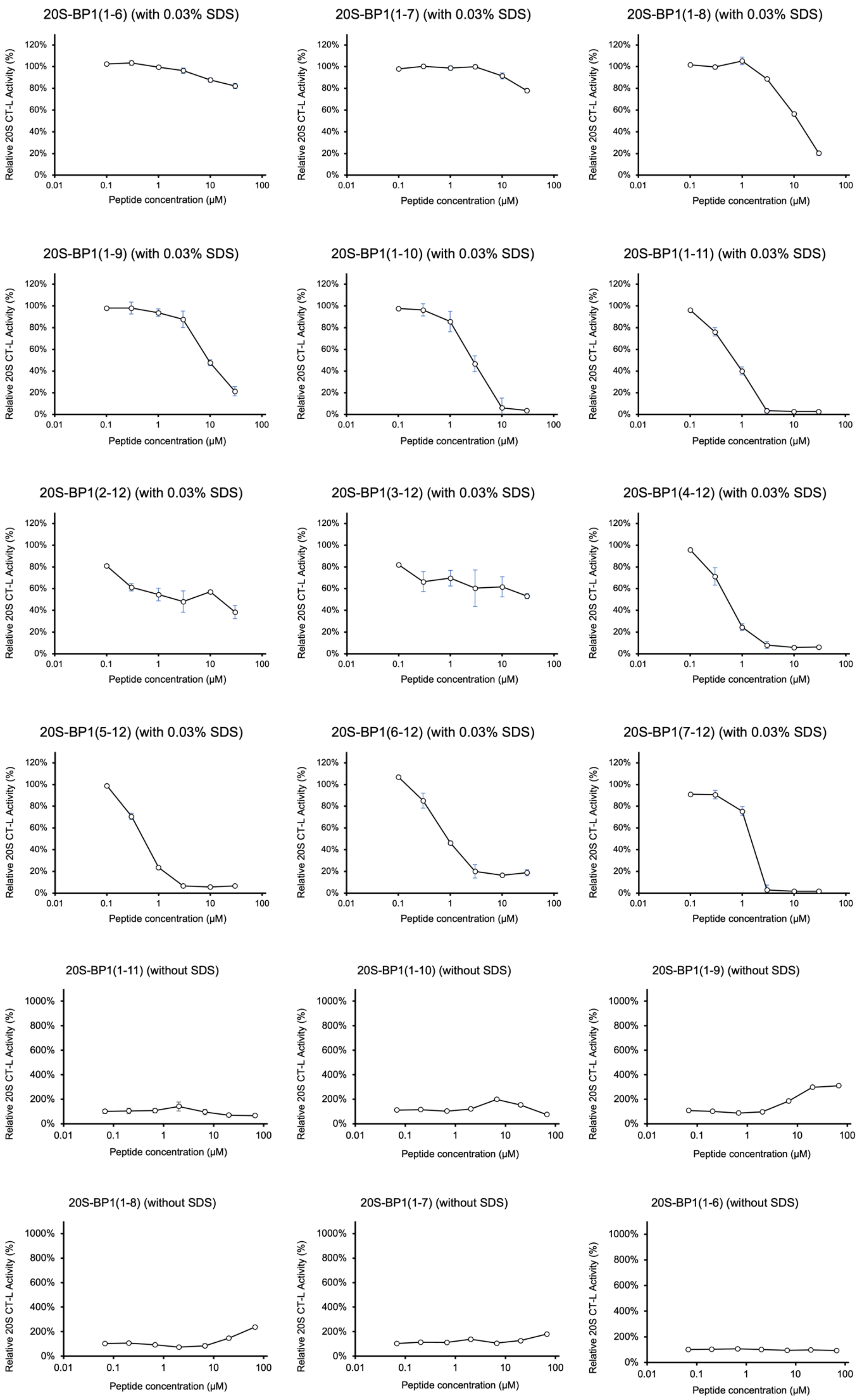
20S-BP1(1-6) (with 0.03% SDS)
20S-BP1(1-7) (with 0.03% SDS)
20S-BP1(1-8) (with 0.03% SDS)
20S-BP1(1-9) (with 0.03% SDS)
20S-BP1(1-10) (with 0.03% SDS)
20S-BP1(1-11) (with 0.03% SDS)
20S-BP1(2-12) (with 0.03% SDS)
20S-BP1(3-12) (with 0.03% SDS)
20S-BP1(4-12) (with 0.03% SDS)
20S-BP1(5-12) (with 0.03% SDS)
20S-BP1(6-12) (with 0.03% SDS)
20S-BP1(7-12) (with 0.03% SDS)
20S-BP1(1-11) (without SDS)
20S-BP1(1-10) (without SDS)
20S-BP1(1-9) (without SDS)
20S-BP1(1-8) (without SDS)
20S-BP1(1-7) (without SDS)
20S-BP1(1-6) (without SDS)
Relative 20S CT-L Activity (%)
Peptide concentration (μM)

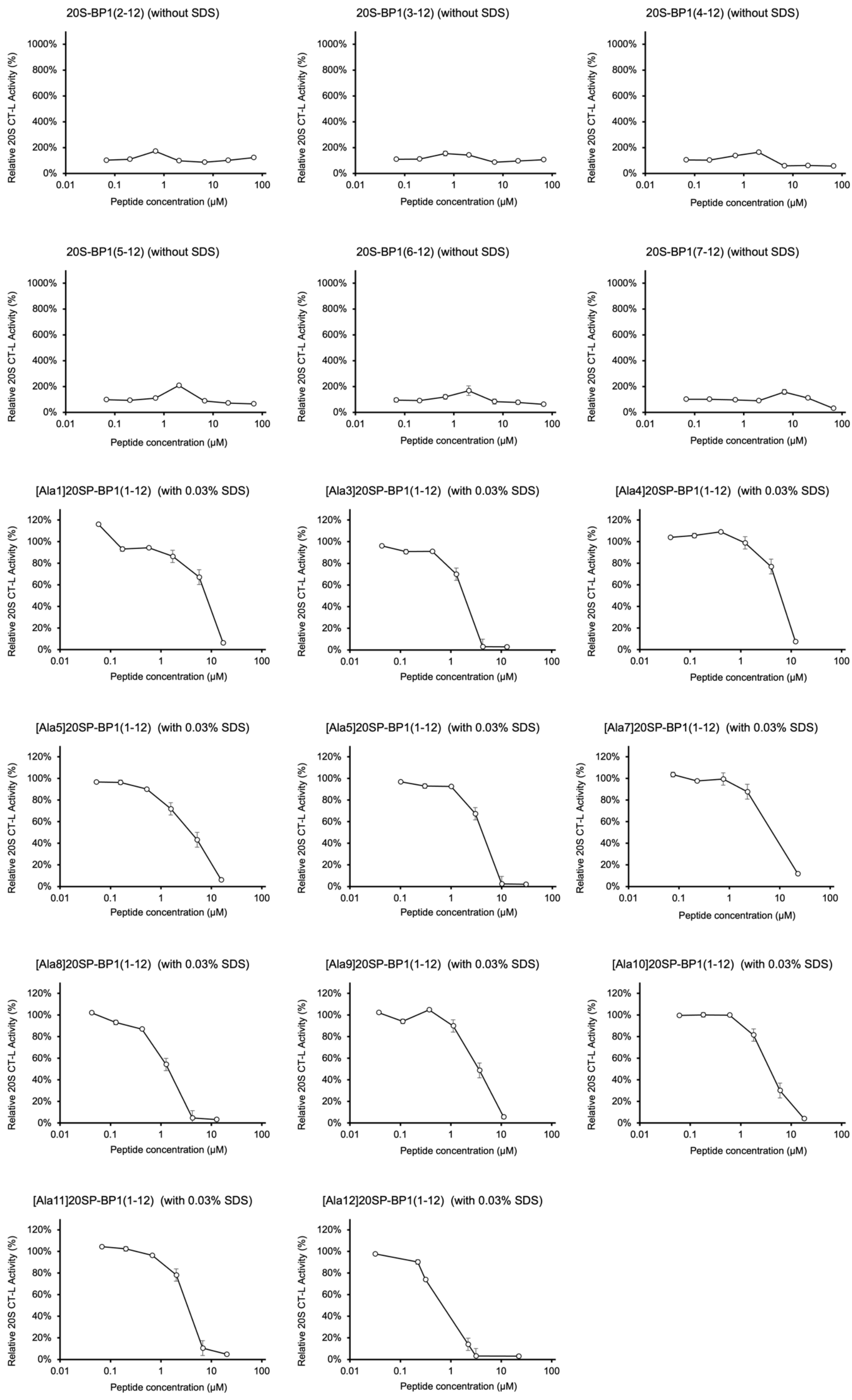
20S-BP1(2-12) (without SDS)
20S-BP1(3-12) (without SDS)
20S-BP1(4-12) (without SDS)
20S-BP1(5-12) (without SDS)
20S-BP1(6-12) (without SDS)
20S-BP1(7-12) (without SDS)
[Ala1]20SP-BP1(1-12) (with 0.03% SDS)
[Ala3]20SP-BP1(1-12) (with 0.03% SDS)
[Ala4]20SP-BP1(1-12) (with 0.03% SDS)
[Ala5]20SP-BP1(1-12) (with 0.03% SDS)
[Ala5]20SP-BP1(1-12) (with 0.03% SDS)
[Ala7]20SP-BP1(1-12) (with 0.03% SDS)
[Ala8]20SP-BP1(1-12) (with 0.03% SDS)
[Ala9]20SP-BP1(1-12) (with 0.03% SDS)
[Ala10]20SP-BP1(1-12) (with 0.03% SDS)
[Ala11]20SP-BP1(1-12) (with 0.03% SDS)
[Ala12]20SP-BP1(1-12) (with 0.03% SDS)
Relative 20S CT-L Activity (%)
Peptide concentration (µM)
0% 200% 400% 600% 800% 1000%
0% 20% 40% 60% 80% 100% 120%
0.01 0.1 1 10 100

20S-BP1(1-8)TYN (with 0.03% SDS)

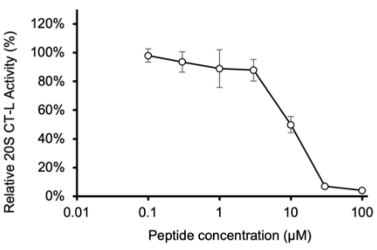


20S-BP1(1-8)LYL (with 0.03% SDS)

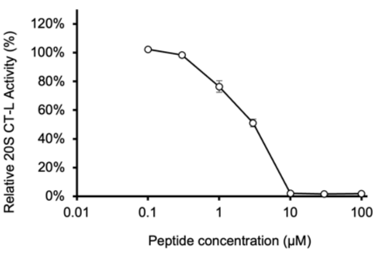


20S-BP1(1-8)FYK (with 0.03% SDS)

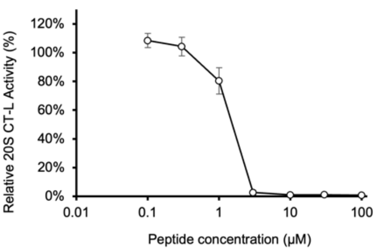


20S-BP1(1-8)KPV (with 0.03% SDS)

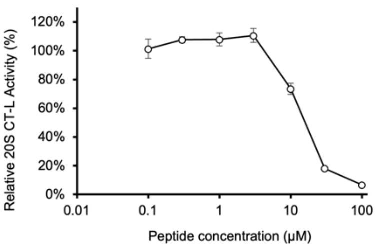


20S-BP1(1-8)LWK (with 0.03% SDS)

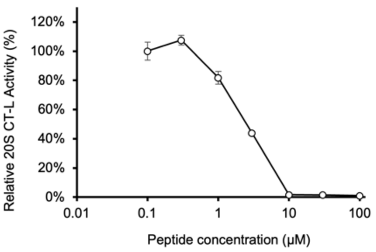


20S-BP1(1-8)TYN (without SDS)

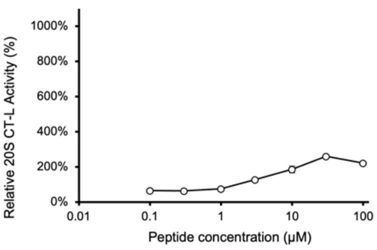


20S-BP1(1-8)LYL (without SDS)

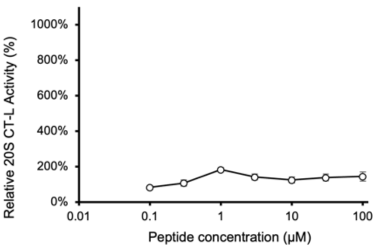


20S-BP1(1-8)FYK (without SDS)

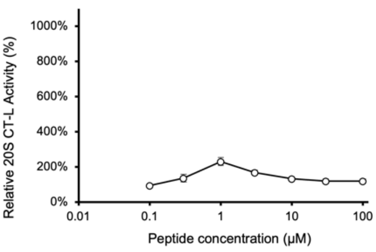


20S-BP1(1-8)KPV (without SDS)

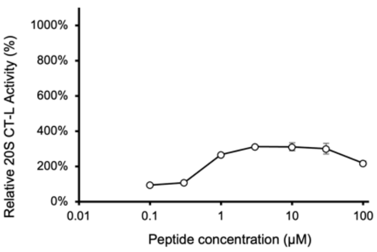


20S-BP1(1-8)LWK (without SDS)

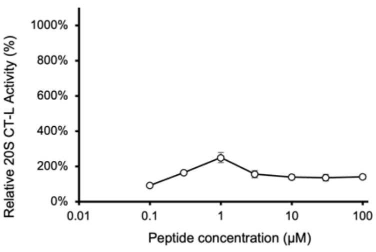


20S-BP1(1-8)YYA (with 0.03% SDS)

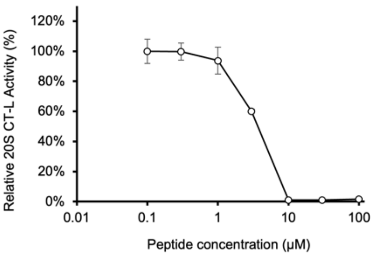


20S-BP1(2-8)YYA (with 0.03% SDS)

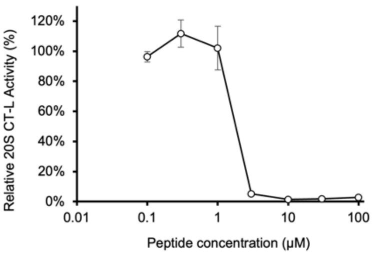


20S-BP1(3-8)YYA (with 0.03% SDS)

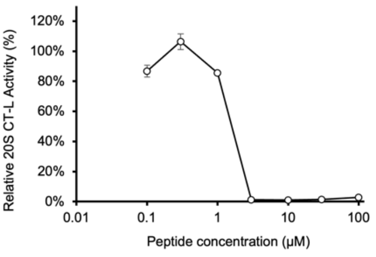


20S-BP1(4-8)YYA (with 0.03% SDS)

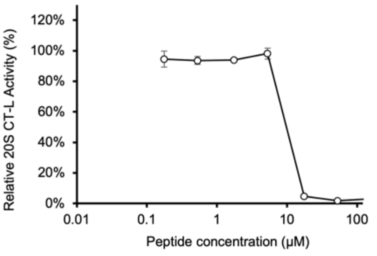


20S-BP1(5-8)YYA (with 0.03% SDS)

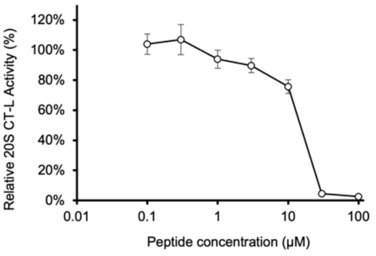


20S-BP1(6-8)YYA (with 0.03% SDS)

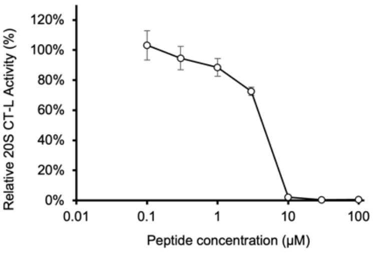

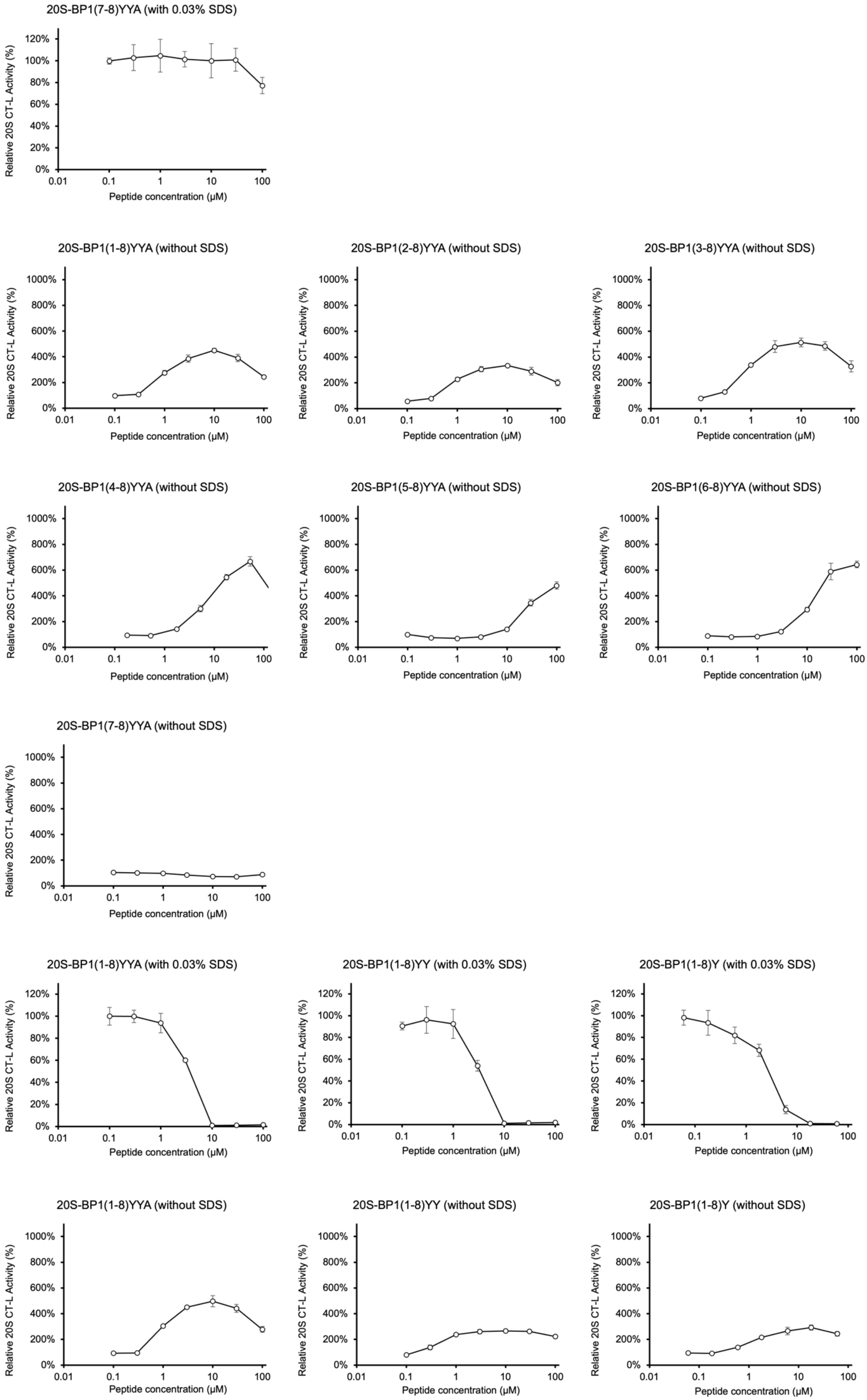
20S-BP1(7-8)YYA (with 0.03% SDS)
20S-BP1(1-8)YYA (without SDS)
20S-BP1(2-8)YYA (without SDS)
20S-BP1(3-8)YYA (without SDS)
20S-BP1(4-8)YYA (without SDS)
20S-BP1(5-8)YYA (without SDS)
20S-BP1(6-8)YYA (without SDS)
20S-BP1(7-8)YYA (without SDS)
20S-BP1(1-8)YYA (with 0.03% SDS)
20S-BP1(1-8)YY (with 0.03% SDS)
20S-BP1(1-8)Y (with 0.03% SDS)
20S-BP1(1-8)YYA (without SDS)
20S-BP1(1-8)YY (without SDS)
20S-BP1(1-8)Y (without SDS)
Relative 20S CT-L Activity (%)
Peptide concentration (μM)
0% 20% 40% 60% 80% 100% 120%
0% 200% 400% 600% 800% 1000%
0.01 0.1 1 10 100

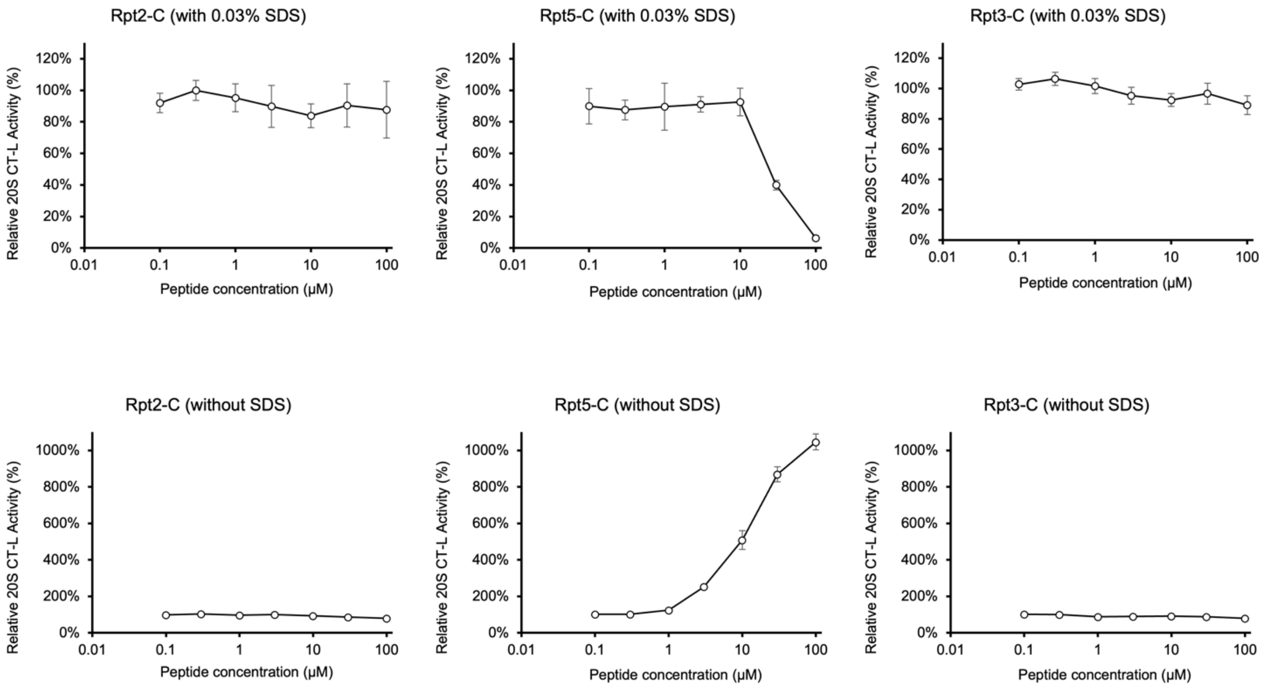


Figure S1. Inhibition (with 0.03% SDS) and promotion (without SDS) of 20S CT-L activity by 20S-BP1 peptides. The fluorescent intensity after 60 minutes incubation with 20S CP (100 μg) and Suc-LLVY-AMC (10 nmol) was defined as 100% (control) in each experiment. The data were collected in triplicate (*n* = 3), and error bars indicate standard deviation.

**Table S1. LC-MS Results**

| Band Number | Protein Name | Mass (Da) | Mascot Score | Matches |
|---|---|---|---|---|
| 1 | PSA6_HUMAN (α1) | 27,838 | 105 | 4 (2) |
| 2 | PSB2_HUMAN (β4) | 22,993 | 63 | 2 (2) |

Protein identification by LC-MS of the two bands is indicated by arrows (Figure 4c).

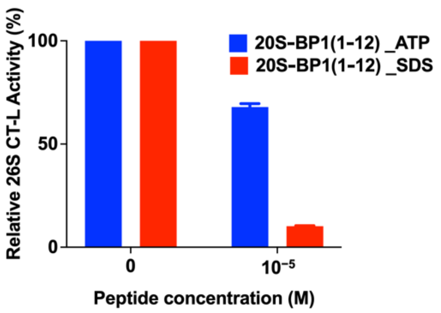


Figure S2. 26S Proteasome fluorometric substrate assay. Inhibition curve of 20S-BP1(1-12) against the 26S proteasome holoenzyme in the presence of ATP (blue closed circles) or 0.03% SDS (red closed triangles).

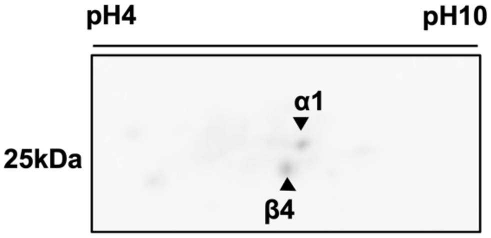


Figure S3. Photoaffinity labeling of the 20S-BP1(5-8)YYA binding site of proteasomes. 2D-PAGE electrophoresis images with photoaffinity labeling detection.

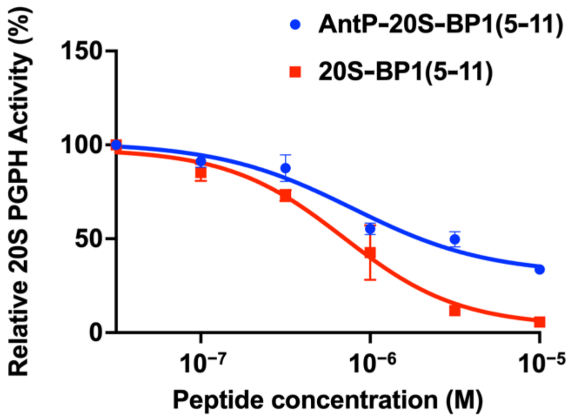

Figure S4. Inhibition (with 0.03% SDS) of the CT-L activity of the 20S CP treated with 0.03% SDS by Antp-20S-BP1(5-11) (blue line) and 20S-BP1(5-11) (red line). Means ± SD, $n$ = 3.

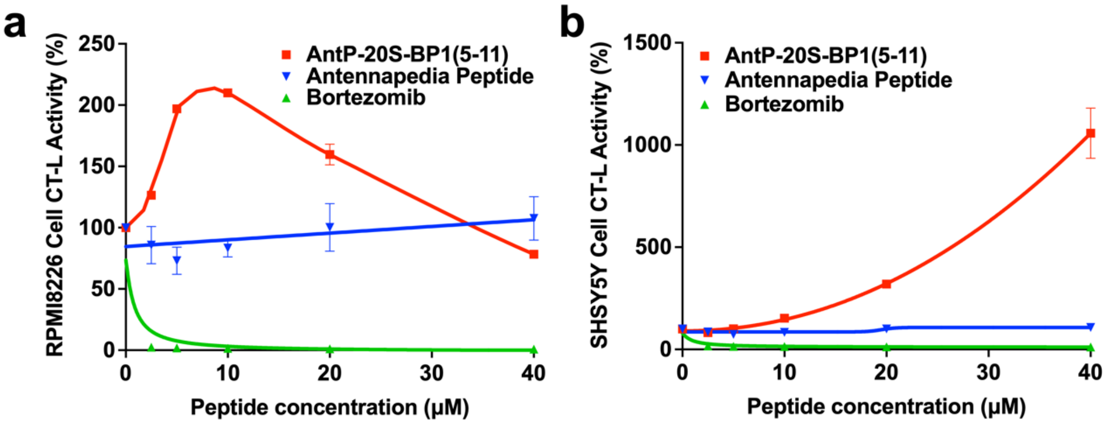


Figure S5. The effects of Antp-20S-BP1(5–11) on RPMI8226 and SHSY5Y human cell lines. (a) Changes of the CT-L activity of the intracellular proteasomes in RPMI8226 cells (a) and SHSY5Y (b) treated with different peptides. AntP-20S-BP1(5–11) (red line); antennapedia peptide (blue line); and bortezomib (green line). Means ± SD, *n* = 3.

Trx-α-syn-20S-BP1(1-8)YYA

MSDKIIHLTDDSFDTDVLKADGAILVDFWAEWCGPCKMIAPILDEIADRYQGKLTVAKLNIDQNPGTAPKY
GIRGIPTLLLFKNGEVAATKVGALSKCQLKEFLDANLAGSGSGHMHHHHHHSSGLVPRGSGMKETAAAK
FERQHMDSPDLGTDDDDKAMADIGSEFLEVLFQGPMDVFMKGLSKAKEGVVAAAEKTKQGVAEAAGK
TKEGVLYVGSKTKEGVVHGVATVAEKTKEQVTNVGGAVVTGVTAVAQKTVEGAGSIAAATGFVKKDQLG
KNEEGAPQEGILEDMPVDPDNEAYEMPSEEGYQDYEPEA**<u>MARPSRLRYYA</u>**

Trx-α-syn-20S-BP1(1-12)

MSDKIIHLTDDSFDTDVLKADGAILVDFWAEWCGPCKMIAPILDEIADRYQGKLTVAKLNIDQNPGTAPKY
GIRGIPTLLLFKNGEVAATKVGALSKCQLKEFLDANLAGSGSGHMHHHHHHSSGLVPRGSGMKETAAAK
FERQHMDSPDLGTDDDDKAMADIGSEFLEVLFQGPMDVFMKGLSKAKEGVVAAAEKTKQGVAEAAGK
TKEGVLYVGSKTKEGVVHGVATVAEKTKEQVTNVGGAVVTGVTAVAQKTVEGAGSIAAATGFVKKDQLG
KNEEGAPQEGILEDMPVDPDNEAYEMPSEEGYQDYEPEA**<u>MARPSRLRHWWR</u>**

Figure S6. Amino acid sequences of the thioredoxin-fused $\alpha$-synuclein recombinant proteins. Red letters indicate enzymatic cleavage sites, and underlined sequences indicate the fused 20S-BP1(1-8)YYA and 20S-BP1(1-12) sequences.

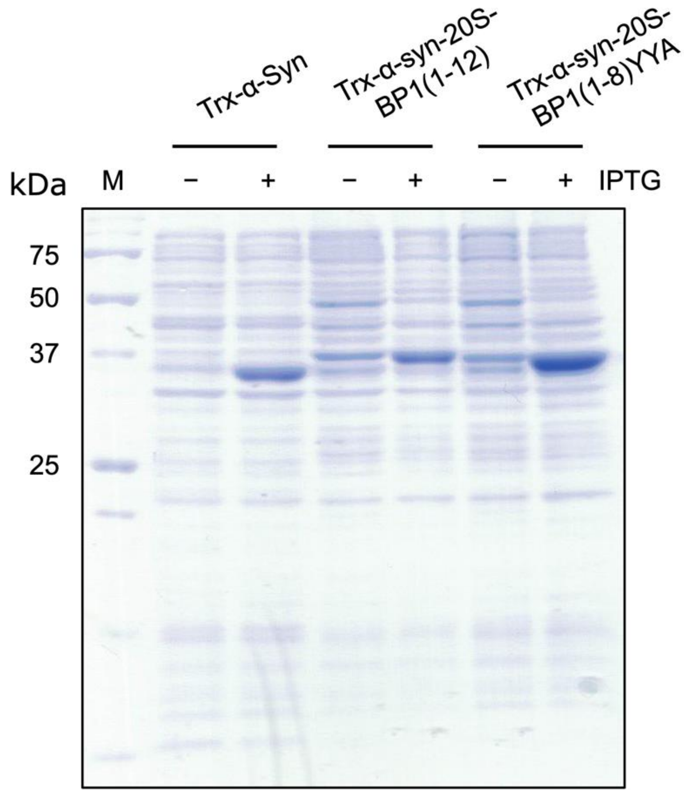


Figure S7. Coomassie Brilliant Blue (CBB)-stained SDS-PAGE images of proteins expressed using the *E. coli* Rosetta (DE3) strain (Novagen). The coding nucleotide sequences of $\alpha$-synuclein and enolase with the 20S-BP1(1-8)YYA sequence fused at the C-terminus (Fig. S5) were inserted into the pET-32a vector.

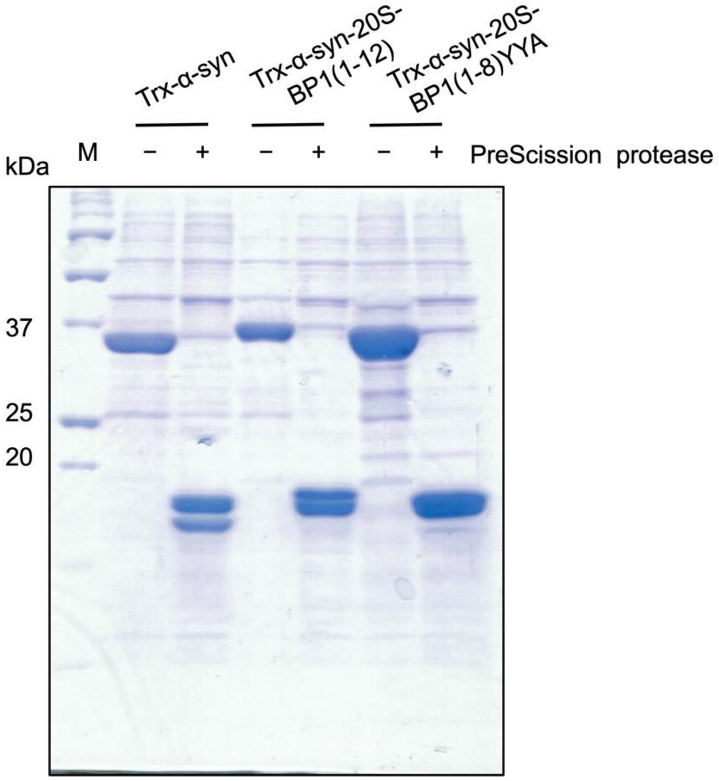


Figure S8. Cleavage of thioredoxin from the target proteins by PreScission Protease (Cytiva). One unit (25 units/μl) of PreScission Protease was added to 100 μg of recombinant protein purified by Ni-chelate chromatography and allowed to react for 24 h at 4°C. Cleaved samples were confirmed by SDS-PAGE.

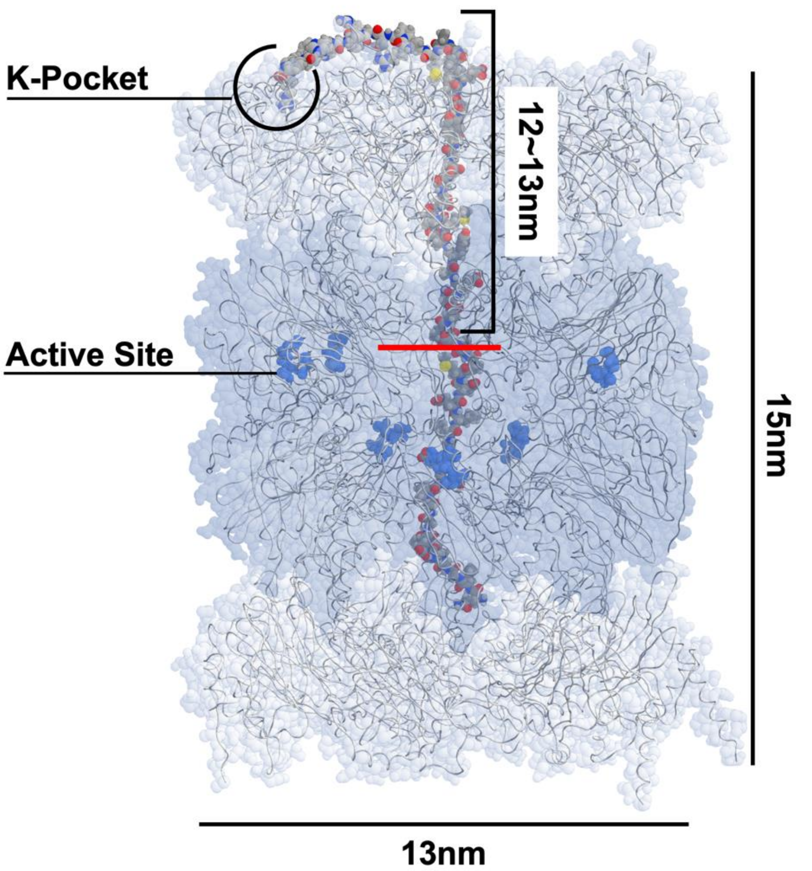


Figure S9. Peptide chain length of $\alpha$-Syn with 20S-BP1 (1-12) from the K-pocket and position of the enzyme active site. Red lines indicate the first cleavage position as shown in Figure 8C.

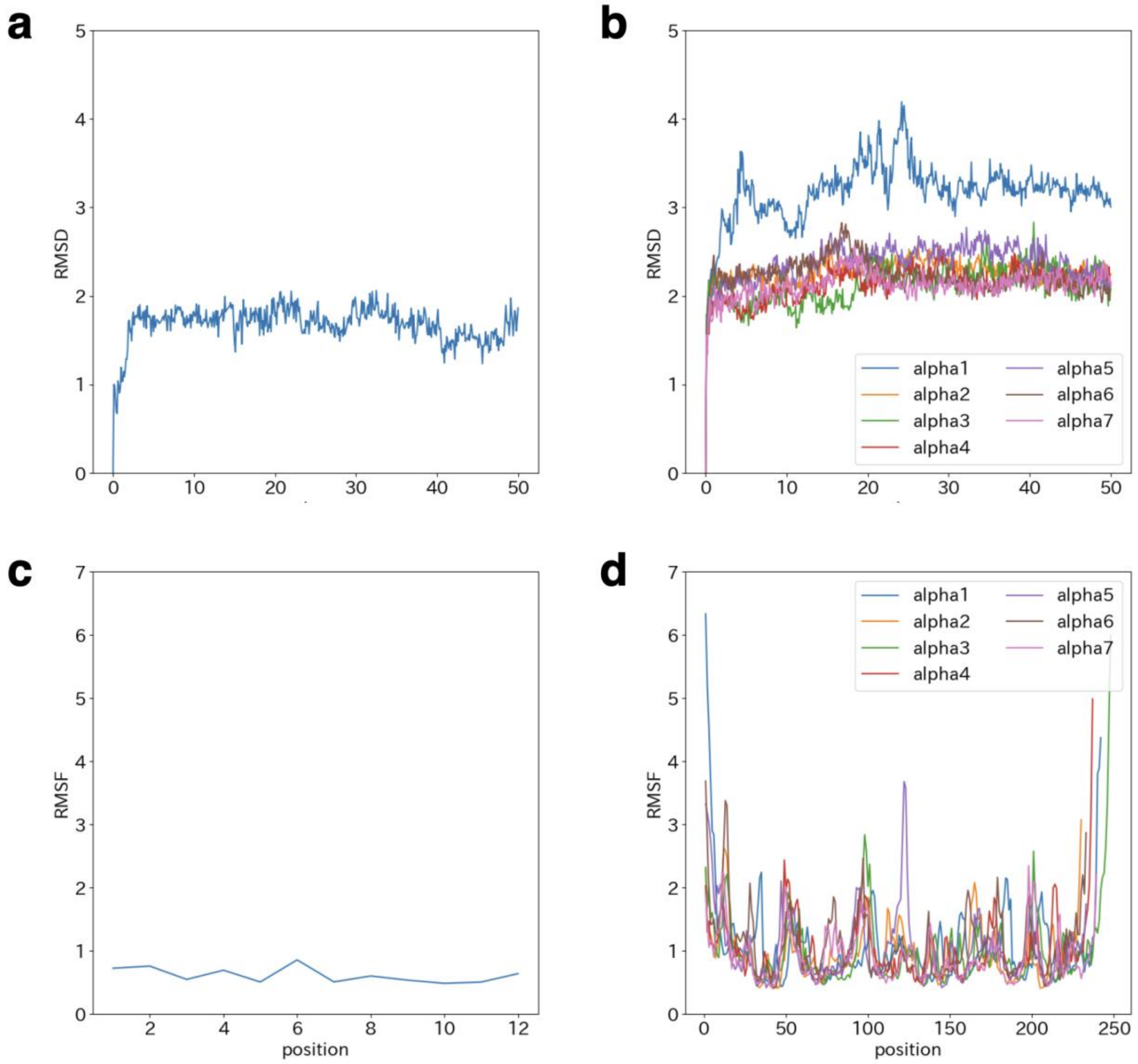


Figure S10. The line plots of RMSD and RMSF values obtained from the MD simulation of the best docking model.

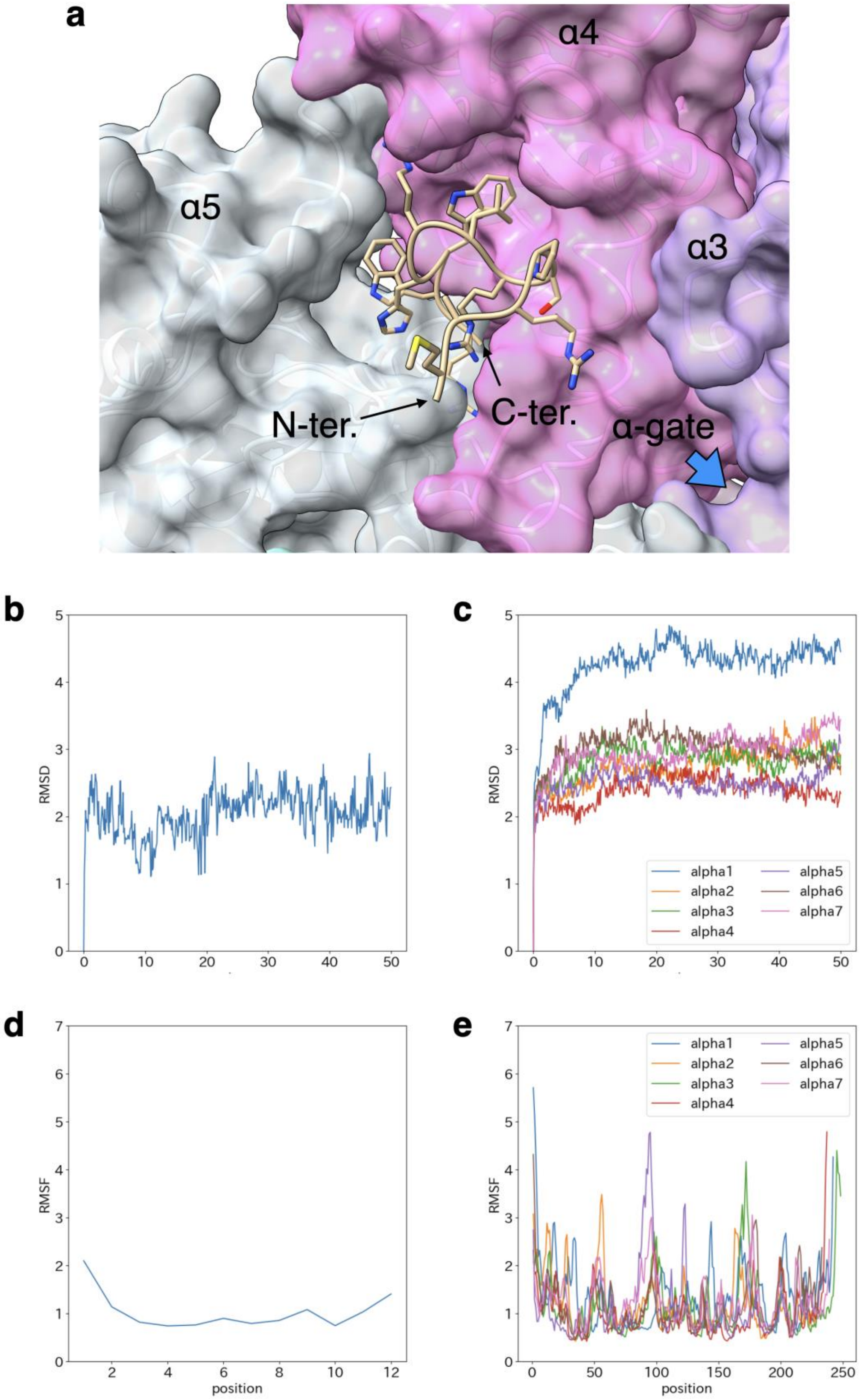


Figure S11. (a) The docking structure of model 2. (b-d) The line plots of RMSD and RMSF values obtained from the MD simulation of the model 2.
